# Sniffing out safety: canine detection and identification of SARS-CoV-2 infection from armpit sweat

**DOI:** 10.3389/fmed.2023.1185779

**Published:** 2023-09-19

**Authors:** Chris Callewaert, Maria Pezavant, Rony Vandaele, Bart Meeus, Ellen Vankrunkelsven, Phaedra Van Goethem, Alain Plumacker, Benoit Misset, Gilles Darcis, Sonia Piret, Lander De Vleeschouwer, Frank Staelens, Kristel Van Varenbergh, Sofie Tombeur, Anouck Ottevaere, Ilke Montag, Patricia Vandecandelaere, Stijn Jonckheere, Linos Vandekerckhove, Els Tobback, Gregoire Wieers, Jean-Christophe Marot, Kurt Anseeuw, Leen D’Hoore, Sebastiaan Tuyls, Brecht De Tavernier, Julie Catteeuw, Ali Lotfi, Alexey Melnik, Alexander Aksenov, Dominique Grandjean, Miguel Stevens, Frank Gasthuys, Hugues Guyot

**Affiliations:** ^1^Center for Microbial Ecology and Technology (CMET), Faculty of Bioscience Engineering, Ghent University, Ghent, Belgium; ^2^Faculty of Veterinary Medicine, Clinique Vétérinaire Universitaire (CVU), University of Liège, Liège, Belgium; ^3^Federal Police Belgium, Linter, Belgium; ^4^CHU Saint-Pierre Hospital, Brussels, Belgium; ^5^CHU-Sart-Tilman, Intensive Care Unit, University of Liège, Liège, Belgium; ^6^CHU-Sart-Tilman, Infectious Diseases – Internal Medicine, Public Health Sciences, University of Liège, Liège, Belgium; ^7^CHU-Bruyères, Intensive Care Unit, University of Liège, Liège, Belgium; ^8^General Hospital (AZ) Glorieux Hospital, Ronse, Belgium; ^9^Onze Lieve Vrouwziekenhuis (OLVZ), Aalst, Belgium; ^10^General Hospital (AZ) Oudenaarde, Oudenaarde, Belgium; ^11^Jan Yperman Hospital, Ypres, Belgium; ^12^Laboratory of Clinical Microbiology, Jan Yperman Hospital, Ypres, Belgium; ^13^HIV Cure Research Center, Department of Internal Medicine and Pediatrics, Ghent University Hospital, Ghent University, Ghent, Belgium; ^14^Department of General Internal Medicine and Infectious Diseases, Ghent University Hospital, Ghent, Belgium; ^15^General Internal Medicine, Clinique Saint-Pierre Ottignies, Ottignies, Belgium; ^16^Namur Research Institute for Life Sciences (Narilis) and Department of Medicine, University of Namur, Namur, Belgium; ^17^General Internal Medicine, Clinique Saint-Pierre Ottignies, Ottignies, Belgium; ^18^Department of Emergency Medicine, ZNA, Antwerp, Belgium; ^19^Belgian Defence, Brussels, Belgium; ^20^Department of Emergency Medicine, ZNA, Antwerp, Belgium; ^21^Respiratory Medicine, GasthuisZusters (GZA) Hospital Group, Antwerp, Belgium; ^22^Emergency Medicine and Intensive Care, GasthuisZusters (GZA) Hospital Group, Antwerp, Belgium; ^23^General Hospital (AZ) Jan Palfijn, Ghent, Belgium; ^24^Department of Chemistry, University of Connecticut, Storrs, CT, United States; ^25^Nosaïs Program, Ecole Nationale Vétérinaire d’Alfort (Alfort School of Veterinary Medicine), University Paris-Est, Maisons-Alfort, France; ^26^Veterinary, Ypres, Belgium; ^27^Department of Surgery, Anesthesiology and Orthopedics of Large Animals, Faculty of Veterinary Medicine, Ghent University, Merelbeke, Belgium

**Keywords:** COVID-19, detection dogs, GC/MS (gas chromatograph/mass spectrometry), acceptability analysis, odor, axilla, vaccination

## Abstract

Detection dogs were trained to detect SARS-CoV-2 infection based on armpit sweat odor. Sweat samples were collected using cotton pads under the armpits of negative and positive human patients, confirmed by qPCR, for periods of 15–30 min. Multiple hospitals and organizations throughout Belgium participated in this study. The sweat samples were stored at −20°C prior to being used for training purposes. Six dogs were trained under controlled atmosphere conditions for 2–3 months. After training, a 7-day validation period was conducted to assess the dogs’ performances. The detection dogs exhibited an overall sensitivity of 81%, specificity of 98%, and an accuracy of 95%. After validation, training continued for 3 months, during which the dogs’ performances remained the same. Gas chromatography/mass spectrometry (GC/MS) analysis revealed a unique sweat scent associated with SARS-CoV-2 positive sweat samples. This scent consisted of a wide variety of volatiles, including breakdown compounds of antiviral fatty acids, skin proteins and neurotransmitters/hormones. An acceptability survey conducted in Belgium demonstrated an overall high acceptability and enthusiasm toward the use of detection dogs for SARS-CoV-2 detection. Compared to qPCR and previous canine studies, the detection dogs have good performances in detecting SARS-CoV-2 infection in humans, using frozen sweat samples from the armpits. As a result, they can be used as an accurate pre-screening tool in various field settings alongside the PCR test.

## Introduction

The SARS-CoV-2 pandemic had dramatic economic and social consequences on a global scale. There was a need for a fast, reliable, inexpensive, easy, non-invasive and widely applicable screening method to distinguish SARS-CoV-2 carriers from non-carriers. Rapid screening and identification of symptomatic as well as asymptomatic and presymptomatic people can contribute to reducing the basic reproduction number of the virus. Even with vaccination efforts, the necessity for fast and reliable detection tools remains crucial to avoid new outbreaks.

Current diagnostic tests are time-consuming, and often come with a considerable cost, while nasopharyngeal swabs are semi-invasive. The predominant method of virus identification, quantitative polymerase chain reaction (qPCR), entails significant time, effort and expense to obtain results. Moreover, the qPCR test gives a considerable amount of false negative results (ranging from 2 to 29%) (1, 2) primarily due to the inherent instability of viral RNA and the potential inadequacy of nasopharyngeal or oral samples in providing enough material. Distinguishing asymptomatic carriers from uninfected individuals can also pose challenges. Additionally, temperature-based screening methods, such as automated forehead temperature sampling, only detect symptomatic people and are inadequate for comprehensive screening purposes.

The human body reacts to the viral infection by producing white blood cells and immune factors. This immune response results in the secretion of various biological molecules and immune factors, some of which are excreted through the skin. Among the regions of the body where these immune factors are notably concentrated is the apocrine sweat regions, particularly the armpits (3). The armpits likewise harbor important lymph nodes that contribute to the production of immune factors. Any bacterial, viral or fungal infection is associated with a unique volatile organic compound (VOC) creation from human cells. These VOCs are subsequently excreted through sweat and are an easy target for rapid screening purposes (4).

Dogs have an extraordinary olfactory capacity and have been successfully used to detect narcotics, explosives, cancer, malaria, metabolic diseases and a wide variety of bacterial and virus infections (5–10). Their trainability using positive reinforcement methods makes them well-suited for detection tasks (11). Common breeds of detection dogs are Belgian Malinois Shepherds, German Shepherds, Cocker Spaniels, Springer Spaniels, Labradors, Pointers, Border Collies, and Beagles. Notably, detection dogs have been employed in diverse settings such as airports, (large) companies, healthcare institutions (hospitals, retirement homes, and triage centers), shopping centers and various mass events including sporting events, cultural gatherings, fairs, concerts and festivals.

Detection dogs are trained to detect SARS-CoV-2 infection based on sweat odor. Previous studies have presented evidence that dogs are able to discriminate SARS-CoV-2 positive from negative samples (12, 13). Preliminary findings show that the dogs were able to discriminate between saliva samples of infected and non-infected individuals with average diagnostic sensitivity of 83% and specificity of 96% (13). Similar preliminary findings, using armpit sweat samples, showed that dogs were able to discriminate with a sensitivity of 83–95% (in 4 dogs) and up to 100% (in another 4 dogs) (12). These encouraging findings form the basis for the development of a reliable screening method for identifying SARS-CoV-2 infected people through the utilization of trained detection dogs.

SARS-CoV-2 detector dogs were trained in many countries around the world, including the United Arab Emirates (UAE), Lebanon, Australia, Chile, Argentina, Brazil, Germany, UK, Finland, France, USA, Russia, Italy, Spain, Colombia, Mexico, Poland, Iran, Peru, Czech Republic, Romania, Canada, Philippines, Switzerland, Saudi Arabia, Austria, Sweden, Georgia, Egypt, Honduras, Tunisia, Bahrain, Singapore, El Salvador and Belgium. A first proof of principle was recently obtained in France (using axillary sweat), Germany (using saliva) and in Finland (using urine). Dogs were trained and could distinguish with a very high success rate positive from negative samples, some of them with up to 100% accuracy (12, 14). SARS-CoV-2 dogs have been deployed in airports and borders in UAE, Lebanon, Saudi Arabia, France, and Finland.

The primary objectives of the present study were as follows:

- To establish a comprehensive biobank consisting of a large sample pool, which would be accessible to all relevant actors in Belgium involved in the training detection dogs. This would facilitate efficient training of the dogs, and ensure the availability of sufficient material for future upscaling.
- To develop a field-testing protocol to train detection dogs in distinguishing between samples infected with SARS-CoV-2 and those that are not infected. This protocol aimed to optimize the dogs’ detection capabilities in real-life scenarios.
- To identify the specific VOCs that the detection dogs are detecting and identify which volatiles make up the characteristic scent observed in SARS-CoV-2 positive sweat samples.

## Materials and methods

### Clinical trials

The protocol has received the approval of Animal Ethical Committee (ULiege, N°20-2246), as well as the approval of the Ethical Committees (Comité d’Ethique Hospitalo-Facultaire University of Liège, approval number 2020/139; Ethical Committee UZ Gent, approval number multicentric study BC-08571, coupled to CHU St Pierre Brussels, AZ Glorieux Ronse (study number TC20/12), AZ Oudenaarde, OLV Hospital Aalst, Jan Yperman hospital Ieper, ZNA hospital (study number 5491), GZA hospitals (study number 210304ACADEM), Jan Palfijn hospital Ghent, AZ Maria Middelares Gent (study number MMS.2021.006), AZ Sint-Vincentius Deinze (study number MMS.2021.006), AZ Jan Portaels Vilvoorde (study number 2021-01), AZ Sint-Lucas Ghent (study number 2020-32), WZC Curando Ruiselede, WZC Armonea, Hospital Saint-Pierre Ottignies) of the different hospitals collaborating to the study. Sweat donors (patients and healthy people) also signed an informed consent at sampling.

### Sweat samples

From October 2020 until April 2021, sampling was organized in Belgium in different hospitals. Positive samples came from CHU-Liege, CHU-ND-Bruyeres, CHU St-Pierre Brussels, St-Pierre Ottignies, UZ Gent, AZ Glorieux Ronse, AZ Oudenaarde, OLVZ Aalst, Jan Yperman Ieper, UZA Antwerp, ZNA Stuivenberg, GZA Anvers, AZ Klina Brasschaat, Jan Palfijn Gent, AZ St-Vincentius Deinze, St-Trudo St-Truiden, AZ Jan Portaels Vilvoorde, AZ Alma Eeklo. Negative samples came from the Kiwanis organization, who organized sampling in different cities in Belgium. Additionally, different care centers (hospitals, senior homes) organized sampling: WZC Armonea Wilrijk, WZC Armonea Spanjeberg, Zorg-Saam WZC Oostakker, WZC Curando Ruiselede, CHU-Liege, CHU St-Pierre Brussels. A list of metadata was collected from each patient/participant, including date, age, biological gender, weight, height, Body Mass Index, ethnicity, postal code, deodorant use, deodorant use frequency, hygiene habits, frequency of underarm washing, medication use, hormonal contraception use, antibiotics use, smoking, comorbidities, (hospital) location of sampling, SARS-CoV-2 symptoms, Ct-value of qPCR result. In the essence of time, dogs used in the present study were trained on a large and diverse set of samples including different hospitals/elderly homes, young and old people, male and female persons, smoker and non-smoker; deodorant user and no underarm cosmetic users.

All sweat donors had their SARS-CoV-2 status (negative or positive) confirmed by qPCR. For positive samples, only patients with clear symptoms (hospitalized) and qPCR results of <30 cycles were preferred. Patients were tested multiple times in the hospital. Patients with no PCR-confirmed test and/or vaccinated against SARS-CoV-2 were excluded. Patients in hospitals with clinical signs related to SARS-CoV-2 (respiratory symptoms, fever) but negative (qPCR) to SARS-CoV-2 were also included. With each donor, a complete but anonymized clinical metadata file was completed. The sweat sampling was performed by trained doctors and/or nurses for safety reasons. It consisted of 5 cotton balls or sterile compresses placed under the 2 armpits of the patient/donor during 15–30 min. For a subset of patients, the sampling was repeated on different days, as long as patients were still SARS-CoV-2 positive. The sampler wore nitrile gloves and a coverall (biological hazard) and handled the samples with a clamp, before putting them in a glass jar or in a closed plastic bag (ziplock). Within 1 h, the plastic bag or the glass jar were frozen (−20°C or colder) and stored until the training of the dogs. Temperature inside the freezer was constantly recorded and was found to be stable.

The tested samples during validation and post-validation were obtained from the original SARS-CoV-2 virus (WIV04 / 2019). At a later stage, samples were obtained from vaccinated people at CHU Saint-Pierre Brussels at least 3 weeks after the second dose of their vaccine (Comirnaty, BioNTech-Pfizer). These samples were also presented to the dogs, together with a positive control sample.

### Dog selection

The dogs were Malinois Shepherds, Border Collie and Springer Spaniel from Federal Police, Civil Security and Army, with previous functions as explosive detection and urban search and rescue. Six dogs were enrolled in the present study up to the validation phase:

- Lilly, Springer spaniel, female, 3 years-old, explosives detector dog, Army
- Xhena, Malinois Shepherd, female, 7 years-old, explosives detector dog, Army
- Tina, Malinois Shepherd, female, 3 years-old, explosives detector dog, Army
- Paxy, Border collie, female, 4 years-old, search and rescue dog, Civil security
- Bailey, Malinois Shepherd, female, 1 year-old, explosives detector dog, Federal Police
- Chaeos, Malinois Shepherd, male, 1 year-old, explosives detector dog, Federal Police.

### Dog training

**Figure 1:**
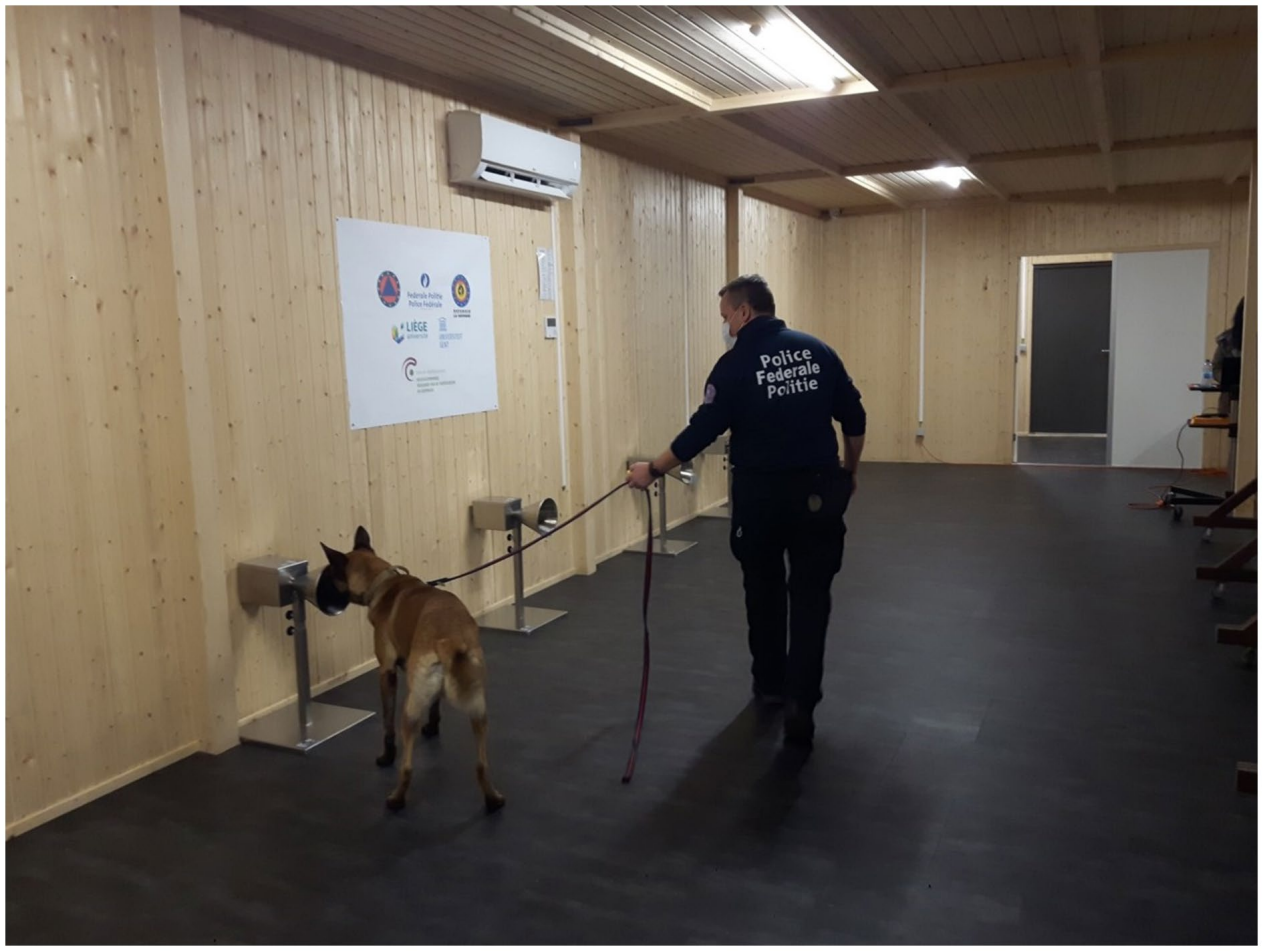
Detection dog (Malinois shepherd) sniffing a sweat sample through a metallic cone during a training at the Training Center of Neerhespen (Federal Police, DACH).

The training took place in Neerhespen (Belgium) at the dog training and accreditation center of the Federal Police (DACH). A spacious room (10 m × 8 m, 80 m^2^) with permanently air-conditioned controlled temperature (16°C) and relative humidity (30%) was used during the training. Metal cones (stainless steel), about 50 cm high from the ground, were used to release the smell of the sweat odors. Behind these cones, a glass jar containing the sweat sample was screwed on and placed in a larger metal box. There was no direct contact between the dog’s nose and the sweat sample (Figure 1).

Sweat samples were removed from the freezer at least 30 min prior to their use. Two cotton balls/compress from one patient were placed into the metal box behind the cones for each dog. The same human patient (2 × 5 cotton balls/compress) could be tested by 2–4 different dogs (2–3 cotton balls per dog per run). After each run (detection of 6–10 samples in a line), the metal cones were wiped off by a cloth soaked in water with 3% of acetone.

Training of the dogs was based on positive reinforcement and classical and operational conditioning principles using primary and secondary reinforcers. When the dog indicates a correct positive sample, a clicker is used to reward the dog. After the click the dog receives a toy (secondary) or food (primary reinforcer). On a negative run the dog gets his toy when there is no false positive indication.

The training was organized in four different steps, beginning mid-December 2020 and ending at the beginning of March 2021 (including validation), for a total of 10 weeks:

1. Odor fixation. On one single cone, dogs sniffed only positive samples of different origins in order to learn how to mark the samples (the dog sits, lies down and/or remains motionless in front of the positive sample). This part lasted 2 weeks.
2. Inclusion of blank samples next to positive samples. Blank samples are compress/cotton balls without sweat. Several cones involved. This part lasted 1 week with about 3 to 4 runs per dog per day.
3. Inclusion of negative samples next to blank and positive samples. Six cones included in the training. This part lasted 2 weeks with about 4 runs per dog per day.
4. Only positive and negative samples, no blank. Six to ten cones were presented in a line to the dogs. This part lasted 3 to 4 weeks with about 4 to 6 runs per dog per day.

After the training, a week (7 days) of validation was organized in the dog center of Neerhespen. This validation was performed in double-blinded conditions whereby neither the dog/handler, nor the second person with the clicker was aware of the number and/or the position of positive samples. The runs consisted of 6 metal cones in line containing either all negative samples, or negative and positive samples (1, 2, or 3 positive samples and the rest negative), but without blank samples. The number of positive and negative samples, as well as the order in the line was randomly attributed for each dog. A same positive sample was systematically tested by 2 dogs, in order to detect any trouble regarding the quality of the sample. The performances of the dogs were calculated after the validation process.

After the validation phase, training continued for 4 more months. Performances after this post-validation phase were also measured and compared to validations’ performances.

### GC/MS analysis and GNPS identification

60 SARS-CoV-2-positive sweat samples, 60 SARS-CoV-2-negative sweat samples and 14 blank samples were analyzed using GC/MS to identify the volatiles present in the sweat samples. The GC/MS analysis was carried out using the Agilent 7200 GC QTOF Agilent Technologies Santa Clara (CA) equipped with a robotic sampler system. The separation was conducted on an HP 5MS column (30 m × 0.25 mm × 0.25 μm). The patch within the vial was heated for 25 min at 200°C to desorb volatiles from the patch and 0.5 mL of headspace injected (injector temperature set at 250°C) into the instrument with a headspace syringe heated to 145°C. The GC protocol analysis included starting temperature 45°C min oven ramp (hold of 2 min), 15°C per min oven ramp to 325°C (hold of 3 min), and 50°C per min oven ramp to 325°C purge the column for reaching equilibration it was 0.2 min for each cycle. The parameters of headspace; Oven, Loop, and Transfer line temperatures were 200°C. Timing parameters for headspacer were 3 min for Vial Equilibration, 0.2 min for Vial Pressurization, and Loop fill, 0.05 min for Loop Equilibration, and 0.2 min for injection. The helium carrier gas was set to constant 1.2 mL per min flow and a splitless injection mode was applied. The purge flow to split vent rate was 50 mL per min at 1 min. The collision gas was N_2_ and collision flow was 1.5 mL per min. Also, the pressure was 9.466 psi and vial pressure was 10 psi. The He gas was used as a quench and Aux gas. The scanned m/z range was 35–400 with the acquisition rate of 10 spectra per second. The empty vial blanks were interspersed with the samples to assess the background signal. Features dataset was normalized. Features dataset were filtered and volatiles present in blank samples were subtracted and removed. Deconvolution and identification of GC/MS spectra was done as described before (15) and using GNPS (16). A PLS DA plot was constructed to understand the distribution of SARS-CoV-2-positive versus SARS-CoV-2-negative samples. Pairwise correlation analyses and random forest analyses were performed to understand differences between positive and negative sweat samples.

### Statistical analysis

This study was held according to the STARD 2015 guidelines (Standards for Reporting Diagnostic Accuracy Studies) (17). The PCR-test was considered in the present study as the “gold-standard” to which our dogs were compared. Sensitivity, Specificity, Accuracy, Positive predictive value, Negative predictive value and Youden index were calculated for each dog, after the validation week and after the post-validation training, using the original formulas (18). The positive and negative samples, as well as the dogs, were randomized beforehand.

### Survey/questionnaire

An online national survey was set up to investigate the overall acceptability toward the use of SARS-CoV-2 detection dogs in practice and distributed through social media, the university websites of UGent and ULiège and through the national press toward the Belgian population. The survey was set up in French and in Dutch. The survey ran from March 5, 2021 till April 19, 2021, with the large bulk obtained in the first week. About 3,591 participants filled in the survey completely. Inclusion and exclusion criteria were residence (only inhabitants of Belgium), language (proficient in Dutch and French). No participants were excluded based on age, sex, race or ethnicity. About 63% of the Belgian population spoke Dutch and 37% spoke French, representing the different language groups in Belgium (Dutch in Flanders, French in Wallonia and both in Brussels). 43.6% of the participants were aged 21-40y, 45.9% were aged 41-65y, 5.4% were younger than 21y and 5.0% were older than 65y. Three quarters of the participants were female (75.3% – or 2,704 of the 3,591) and one quarter was male. Results were combined and translated to English and analyzed in R. To identify main correlations between results of questions, correlation analyses and random forest analyses were performed.

## Results

### Sampling and optimal training protocol

The present study used the standard protocol developed in France as foundation for its research (12). The implementation of this protocol required adjustments due to encountered challenges. Initially, 15 detection dogs from different organizations, specialized in detecting explosives and human search tasks, were included in the study. However, not all dogs were suitable for this study, leading to a reduction of participating dogs. Some of the Urban Search & Rescue dogs were excluded due to difficulties encountered when working with cones positioned in line. The availability of sufficient positive and negative sweat samples was also crucial to allow an in-depth high frequency training of the dogs. The training regimen required several runs per day, with each dog undergoing up to 300 and more runs for a complete training. Initially, reuse of samples was done but was abandoned to avoid undesirable imprinting of specific samples in the dogs. The number of dogs involved in the study was reduced to 6, in order to increase the frequency of runs from 3 to 6 runs per day. A 5 day per week schedule was followed. The availability of a substantial number of positive and negative sweat samples was crucial, considering the high turnover of these samples. Additionally, efforts were made to improve the cleaning process of the cones between runs as well as to enhance the practical organization of the runs, including a correct recording of all results. These adjustments aimed to optimize the training process and manage the practical aspects of the study effectively.

### Dog training results during and after validation

The validation process involved the utilization of 397 positive samples from 51 different patients and 1,629 negative samples collected from 276 volunteers. Each dog underwent the task of detecting an average of 66 ± 11 (Mean ± SD) positive samples and 272 ± 28 negative samples during the validation phase. This consisted a total of 58 ± 6 runs per dog over a 7-day period. The individual presentation of samples per dog as well as the Sensitivity, Specificity, Accuracy, Positive predictive value, Negative predictive value and Youden index of each dog after validation are represented in Table 1. Further individual details of the sample detection by the dogs can be found in Supplementary Table S1. The combined performance of all dogs yielded an overall specificity of 98% and an overall sensitivity of 81%. The overall accuracy amounted to 95%. The performance of the test, all dogs combined, evaluated with the Youden index was almost 80%.

**Table 1:** Diagnostic performances of the six detection dogs after validation.

Dog	Se* %	Sp* %	PPV* %	NPV* %	Youden %	Accuracy %	N Run*
Paxi	88	100	100	97	88	98	51
Cheos	81	99	95	95	80	95	61
Xhena	94	96	83	99	90	96	50
Tina	73	95	78	94	68	91	56
Lilly	76	100	100	94	76	95	63
Bailey	76	100	98	95	76	95	65
Total	81	98	92	95	79	95	58 ± 6

*Se, sensitivity; Sp, specificity; PPV, positive predictive value; NPV, negative predictive value; N, number.

The 6 same dogs were involved in continued training after the validation phase for 4 months (approximately one training every 2 weeks), from mid-March 2021 until the end of May 2021. They went to 6 training sessions of one-day each, with a total of 48 ± 6 runs per dog, and thus an average of 9 ± 2 runs per dog per training. The dogs were confronted to about 396 negative samples and 46 positive samples. During the post-validation phase, the dogs had to test different patterns of cone distribution: 5 to 10 negatives with all negatives, or a combination of 1 to 3 positive with 5 to 10 negatives. Table 2 summarizes the performances of the 6 dogs after the post-validation phase. The overall specificity and sensitivity were 99 and 80%, respectively. The performance of the test (Youden index) was almost 80%. The performances of the dogs at validation and after validation are not significantly different (*p* > 0.1).

**Table 2:** Performances of the six dogs at the end of the post-validation phase (six trainings).

Dog	Se* %	Sp* %	PPV* %	NPV* %	Youden %	Accuracy %	N* Run
Paxi	88	100	97	98	88	98	49
Cheos	96	99	93	99	95	99	51
Xhena	78	99	96	96	78	96	58
Tina	76	98	85	96	74	95	44
Lilly	73	100	100	95	73	96	40
Bailey	70	100	100	95	70	96	46
Total	80	99	95	97	79	97	48 ± 6

*Se, sensitivity; Sp, specificity; PPV, positive predictive value; NPV, negative predictive value; N, number.

### Dog results on vaccinated patients’ samples

Sweat samples were obtained from 28 vaccinated people, who received the Comirnaty vaccine (BioNTech-Pfizer), in two doses and were sampled (armpit perspiration, cotton pads frozen at −20°C before training) 3 weeks after the second dose. These samples were tested by five dogs during training. The vaccinated samples were mixed along with other negative (unvaccinated) and positive (SARS-CoV-2) samples (each run included at least 6 different samples). Each dog performed 2 ± 1 runs (minimum 1 run, maximum 4 runs). The overall performances showed Se 77%, Sp 100%, and Youden index 0.77 (Supplementary Table S3). When taking into account only the vaccinated samples, the dogs considered the samples as negative in 100% of the cases.

### Effect of age, biological gender, body mass index, deodorant use, medication use and sample location on detection by the dogs

**Figure 2:**
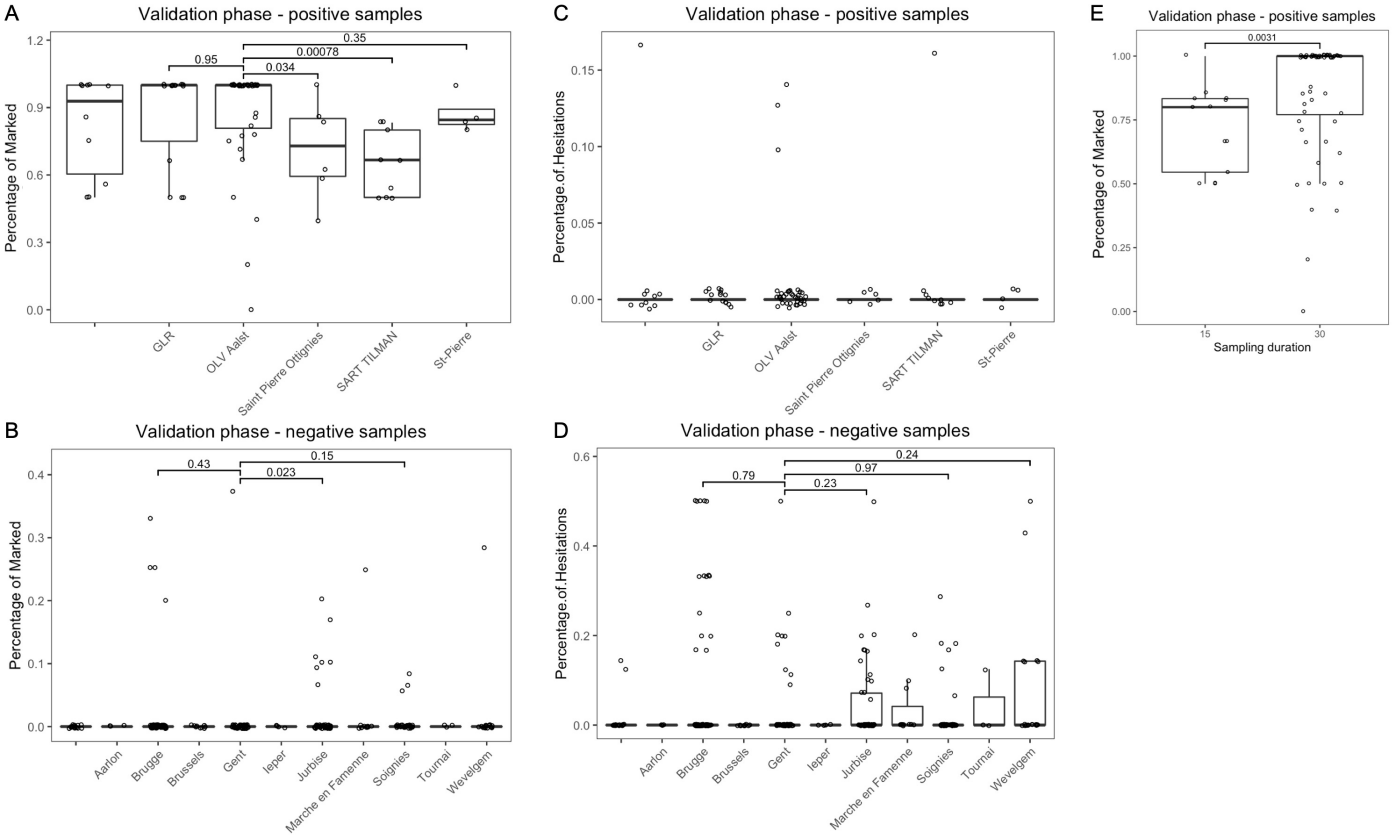
Impact of sampling location and sampling time on marking by the six trained SARS-CoV-2 detection dogs during validation phase. **(A)** Impact of sampling location on percentage of marking by the detection dogs in the SARS-CoV-2 positive samples (from hospitals). **(B)** Impact of sampling location on percentage of marking by the detection dogs in the SARS-CoV-2 negative samples (from volunteers). **(C)** Impact of sampling location on percentage of hesitations by the detection dogs in the SARS-CoV-2 positive samples (from hospitals). **(D)** Impact of sampling location on percentage of hesitations by the detection dogs in the SARS-CoV-2 negative samples (from volunteers). **(E)** Impact of sampling duration on percentage of marking by the detection dogs in the SARS-CoV-2 positive samples (from hospitals).

Sampling location, and more importantly, sampling time were important influencing factors in the detection rate by the detection dogs. We found significant differences in marking by the detection dogs based on sampling location and sampling time (Figure 2). A shorter sampling time resulted in a lower detection rate by the detection dogs. Of the 13 samples that were taken from SARS-CoV-2-positive patients in hospitals that were held for only 15 min, instead of 30 min, only 1 sample was marked as positive by all six detection dogs. A sampling time of 30 min resulted in a significantly higher detection rate by the dogs (*p* = 0.0031). A shorter sampling time of 15 min was employed at CHU St-Pierre in Brussels and CHU Sart-Tilman Liège. The percentage of marking by the detection dogs was lower for these two hospitals (*p* = 0.0078 for CHU Liège as compared to Hospital OLV Aalst, where sampling was done for 30 min). The Saint-Pierre hospital in Ottignies also showed lower detection rates as compared to OLV Aalst (*p* = 0.034). A different sampling method or incomplete understanding of the sampling protocol could also affect the detection rate by the dogs.

There was no significant correlation found between the age of the participants providing the samples and the dogs’ detection (or hesitation), although the age of the participants significantly differed between SARS-CoV-2 positive and negative samples (Supplementary Figure S1). The biological gender of the patients or volunteers did not influence the marking or hesitation by the six trained SARS-CoV-2 detection dogs, in either the SARS-CoV-2 positive or negative group (Supplementary Figure S2). A series of other variables were tested on their potential correlation with the detection rate by the detection dogs. Body mass index (BMI) had no influence on the marking by the six trained detection dogs during the validation phase (Supplementary Figure S3). The BMI was comparable among the SARS-CoV-2 positive and negative samples. Deodorant use similarly did not impact the marking by the six trained detection dogs during the validation phase. No significant differences were found between samples coming from people that either did or did not use deodorant. Medication use by patients or volunteers was tested and found to have a significant correlation with the marking percentage by the detection dogs. Surprisingly, the number of markings on the negative samples was higher if volunteers reported to not have used medication. This is likely a confounding factor to the sampling location, as the full survey completion varied across locations.

### GC/MS

**Figure 3:**
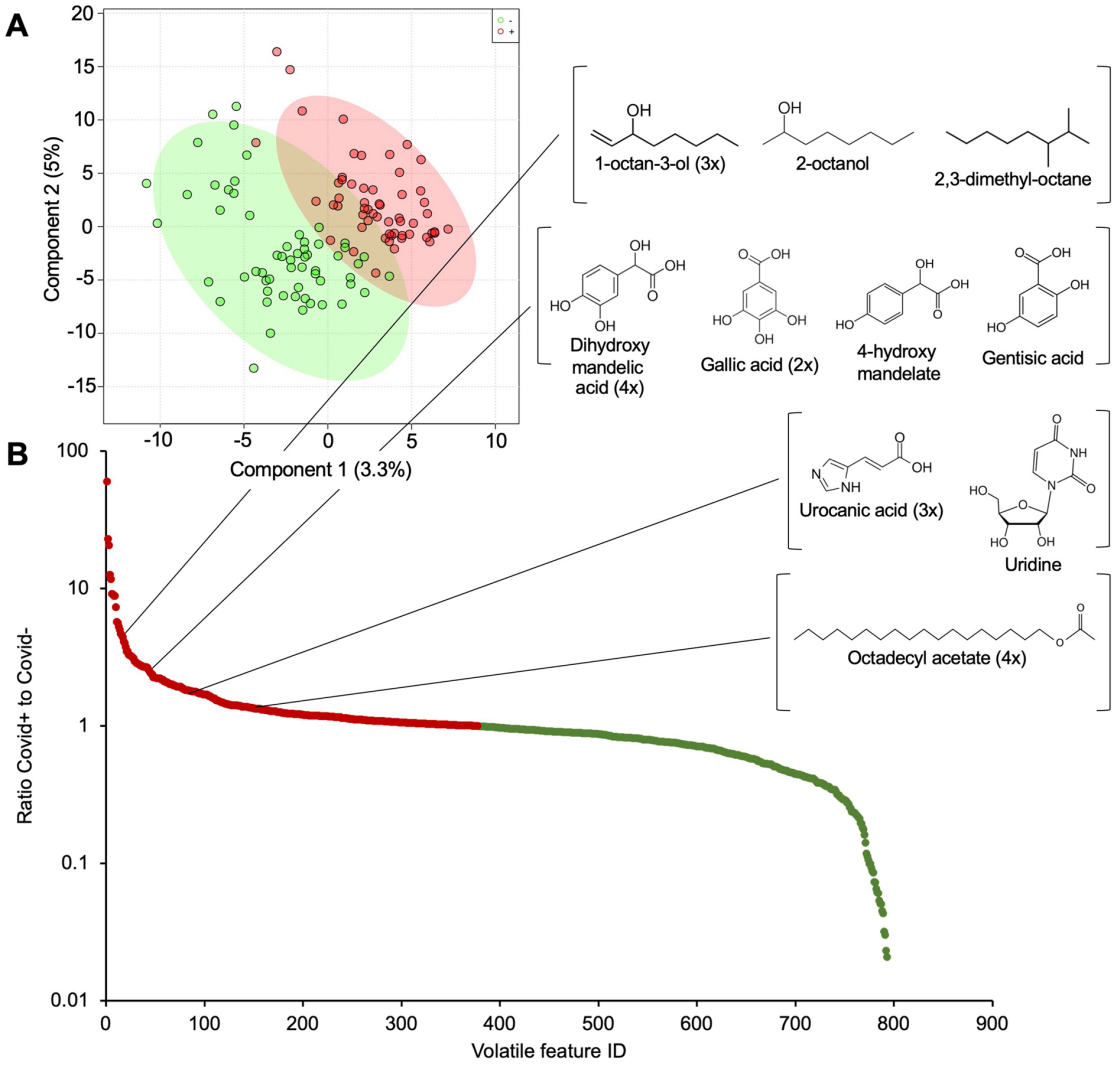
Molecular exploration of differences between SARS-CoV-2-positive and SARS-CoV-2-negative sweat samples. **(A)** PLSDA plot indicates clear differences in metabolic background among the SARS-CoV-2-positive (red) and SARS-CoV-2-negative (green) sweat samples. **(B)** The plot shows the ratio of abundances of volatiles in SARS-CoV-2-positive versus SARS-CoV-2-negative sweat samples. Ratios higher and lower than one (i.e., volatiles that are enriched in SARS-CoV-2+ versus SARS-CoV-2- samples) are highlighted in red and green, respectively. Examples of important and recurrent annotated volatiles and their respective molecular related volatiles are depicted.

This study aimed to identify the volatiles that are detected by the dogs and marked as positive. Our results indicate that SARS-CoV-2-positive samples indeed contained different signature volatiles that were significantly less present in SARS-CoV-2-negative sweat samples (Figure 3A). The detection dogs did not pick up one single compound, but rather a wide variety of different volatiles (Figure 3; Table 3; Supplementary Figure S4). Several classes of volatiles were repeatedly found as significantly enriched in SARS-CoV-2-positive as compared to SARS-CoV-2-negative samples. Five volatiles structurally related to 1-octan-3-ol were significantly associated with positive sweat samples, and were not found in SARS-CoV-2-negative samples (Figure 3B and Table 3). Seven volatiles which were structurally related to DL-3,4-dihydroxymandelic acid, and its metabolites, were similarly associated with positive sweat samples (Figure 3B and Table 3). Urocanic acid and its metabolites were detected several times and significantly linked to SARS-CoV-2-positive samples (Figure 3B and Table 3). Octadecyl acetate and its derivatives were another important group of volatiles detected in the positive samples (Figure 3B and Table 3). These and a series of other (unknown) volatiles form the unique scent that the detection dogs picked up and assigned as positive sweat samples.

**Table 3:** SARS-CoV-2-positive associated volatiles, with *p*-value and putative feature identity.

Retention time	Covid	*p*-value	Feature ID
2.03	+	7.61E-06	1-octen-3-ol
2.46	+	0.0045	2-octanol
5.60	+	0.0013	1-octen-3-ol
5.60	+	0.0413	1-octen-3-ol
16.11	+	0.0386	2,3-dimethyl-octane
4.66	+	0.0094	Gallic acid
5.94	+	0.0002	Gallic acid
6.45	+	0.0103	4-Hydroxymandelate
6.57	+	0.0317	DL-3,4-Dihydroxymandelic acid
6.65	+	0.0418	Gentisic acid
6.95	+	3.24E-05	DL-3,4-Dihydroxymandelic acid
7.98	+	0.0007	DL-3,4-Dihydroxymandelic acid
9.28	+	0.0002	Urocanic acid
9.28	+	0.0227	Urocanic acid
9.82	+	0.0073	Urocanic acid
10.23	+	6.16E-05	Uridine
11.99	+	0.0465	Octadecyl acetate
12.11	+	0.0012	Octadecyl acetate
12.17	+	0.0251	Octadecyl acetate
12.50	+	0.0177	Octadecyl acetate
3.22	+	0.0360	2-methyl-N-ethyl-N-octadecyl-propanamide
3.32	+	0.0079	2-methyl-N-ethyl-N-octadecyl-propanamide
3.76	+	0.0004	Unknown
4.11	+	0.0454	Hexanal
4.53	+	0.0053	3-Acetoxy-2-chlorpromazine
7.01	+	0.0112	N-phenyl-benzenemethanamine
8.62	+	0.0208	Camphor
8.72	+	0.0113	Camphor
10.79	+	0.0251	trans-2-tert-butyl cyclohexanol acetate
11.33	+	0.0218	L-Ascorbic acid
12.69	+	0.0019	(6Z,9Z)-6,9-Hentriacontadiene
12.95	+	0.0248	(6Z,9Z)-6,9-Hentriacontadiene
13.28	+	0.0294	3,4-Dihydro-2,5,7,8-trimethyl-2-benzyloxycarbonyl-2H-1-benzopyran-6-ol
16.74	+	0.0335	N-hexyl-acrylamide
18.14	+	0.0205	18-Nonadecenoic acid
20.65	+	0.0341	Tetradacanal
21.87	+	0.0213	unknown

### Acceptability results of SARS-CoV-2 detection dogs

**Figure 4:**
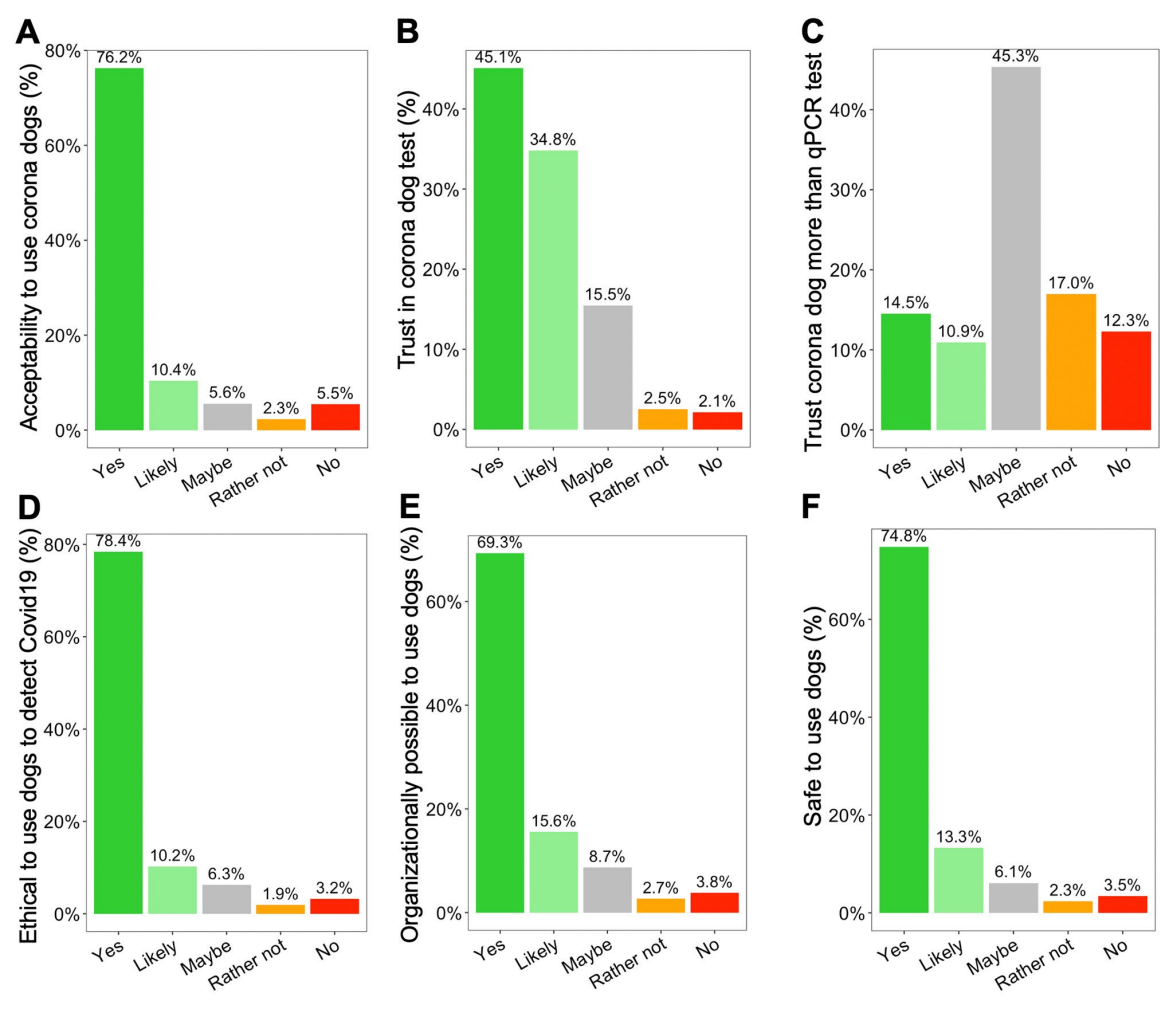
Results from the national survey on acceptability toward the use of SARS-CoV-2 detection dogs in practice. **(A)** Do you accept detection dogs to be used for this purpose? **(B)** Do you trust in the outcome of the SARS-CoV-2 detection dogs? **(C)** Which test would people trust more: the outcome of the SARS-CoV-2 detection dogs or the outcome of the qPCR test? **(D)** Is it ethical to use detection dogs for this purpose? **(E)** Is it organizationally possible to use detection dogs in real life? **(F)** Is it dangerous to use dogs for this purpose?

A large majority of the responders (76.2%) fully agree that dogs can be used to diagnose SARS-CoV-2 infection (Figure 4A). And an even larger majority (81.2%) of the responders fully agree that dogs can be used to diagnose SARS-CoV-2 infection based on a sweat sample (results not shown). The outcome of the corona dog outcome was similarly trusted by the responders (45.1%) (Figure 4B). About 34.8% would likely trust the outcome and 15.5% would maybe trust the outcome of the detection dog. However, there were still some doubts among the trustworthiness, mostly as the qPCR test result was trusted better (*p* < 0.001). Still, if we asked which test would be trusted more, the majority of the responders (45.3%) did not know which test would be the most trustworthy: the qPCR test result or the corona dog test result (Figure 4C).

The large majority (78.4%) of the responders did not have any ethical problems with the use of detection dogs to trace SARS-CoV-2 with people (Figure 4D). Nonetheless, 21.6% of the responders had some form of ethical questions around the use of dogs for this purpose. The ethical concerns were the major confounding factor in the general acceptance of the corona dogs in practice (*p* < 0.001). It was also a main confounding factor in the general trust in the outcome of the SARS-CoV-2 detection dogs (*p* < 0.001) (Supplementary Figure S5A). This suggests that the ethical considerations surrounding the use of detection dogs played a pivotal role in shaping both the acceptance and trust as a detection method. The majority (69.3%) of the responders expressed no concerns in the practical organization of the SARS-CoV-2 detection dogs to sniff SARS-CoV-2 infection with people (Figure 4E). There were nonetheless 30.7% of the responders that did have some doubts on the logistical aspects of employing SARS-CoV-2 detection dogs. These practical doubts were also a main confounding factor in the general acceptance of the corona dogs in practice (*p* < 0.001) (Supplementary Figure S6A).

The large majority (74.8%) of the responders found it safe to use dogs for the purpose of detecting SARS-CoV-2 infection with people (Figure 4F). There were however still some doubts on the safety of using sniffer dogs and it was the second biggest confounding factor in the general acceptance of SARS-CoV-2 detection dogs in practice (*p* < 0.001) and the second biggest confounding factor in the general trust in the outcome of the SARS-CoV-2 detection dogs (*p* < 0.001) (Supplementary Figure S5C). Fear of dogs was a confounding factor in the general acceptability (*p* < 0.001) and in the general trust of the outcome of the detection dog (*p* < 0.001). A dog allergy or religious problems with dogs did not have any significant relationship with the general acceptability in the SARS-CoV-2 detection dogs (*p* = 0.8385 and *p* = 0.6636, respectively).

A large majority (77.8%) was willing to donate their armpit sweat for the purpose of training SARS-CoV-2 detection dogs. The willingness to share their armpit sweat to train detection dogs was also a confounding factor in the acceptance of the responders (*p* < 0.001). However, when asking which sample the responders would be most likely to give for the SARS-CoV-2 detection dog to evaluate, the majority indicated they would rather give a sample of their armpit sweat (64.2% of the responders) than a nasal sample (24.3% of the responders). Other samples that the responders are willing to provide are their mouth mask (70.0% of the responders) and a sample of their saliva (66.9% of the responders). However, when needing to take a decision on which test to take when arriving at the airport, most people preferred the dog test (60.6%) or the dog test together with a qPCR test (27.7%) over the qPCR test (4.6%) or another fast test (7.1%) (Supplementary Figure S6B). The responders indicate the unpleasant feeling that is associated with taking a sample for a qPCR test and the convenience of providing a sample for the SARS-CoV-2 detection dog to evaluate.

Communication on the SARS-CoV-2 detection dogs played a significant role in influencing the general acceptability and trust in the SARS-CoV-2 detection dogs (Supplementary Figure S5B). Respondents who had prior exposure to information about these detection dogs, through press and social media, exhibited higher levels of acceptance (*p* < 0.001), with communication resulting in a 30% increase in acceptability for practical implementation. Communication also had a significant impact on the general trustworthiness of the SARS-CoV-2 detection dogs (*p* < 0.001). The age of the responders also played a significant influence on the overall acceptability and trust in the detection dogs (Supplementary Figure S5D). Younger age groups displayed lower levels of acceptance (*p* < 0.001) and general trustworthiness in the outcome of the detection dogs (*p* < 0.001), including concerns related to potential refusal at the border or entrance of an event based on the result of SARS-CoV-2 detection dogs (*p* < 0.001). When asked about the location where to deploy the corona dogs, the responders preferred to use them in the airport (88.4% of the responders), cultural events (78.0% of the responders), and sports events (70.9% of the responders) (Supplementary Figures S6C,D). The language group (2,261 Dutch speaking and 1,330 French speaking) did not have any influence in the general acceptability or trust in the SARS-CoV-2 detection dogs. The biological gender also did not have a significant correlation with the general acceptance on the use of SARS-CoV-2 detection dogs, nor in the general trust in the outcome of the detection dogs.

## Discussion

The trained SARS-CoV-2 dogs demonstrated an overall accuracy of 95% after the validation phase. The average sensitivity, measuring the ability to correctly identify positive cases, was 81%, while the average specificity, indicating the ability to correctly identify negative cases, was 98%. The performance measures remained consistent during the post-validation stage, with an average sensitivity of 80% and average specificity of 99%. These results are in accordance with the general recommendations set by the European Center for Disease Prevention and Control and the World Health Organization, thereby requiring minimum 80% sensitivity and minimum 97% specificity for a valid SARS-CoV-2 test. Therefore, the SARS-CoV-2 detection dogs can be considered as reliable rapid antigen tests.

The results presented in the validation and post-validation phase are comparable with those of other studies. Other research groups have used different samples to train the detection dogs (19): 1/ armpit sweat (12–14, 20–22), or 2/ saliva or tracheobronchial secretion (13, 23, 24), or 3/ urine (13), or 4/ masks and clothes (24). Overall sensitivity and specificity, with all types of samples included, varied from 65 to 100%, and from 85 to 98%, respectively. While comparing studies working only with sweat samples, the sensitivity ranged from 71 to 100%, and the specificity ranged from 85 to 99%. We attempted to document the qPCR cycle threshold (Ct value) for samples included; however, this was not possible for each sample used throughout the study. We observed that the dogs could more easily detect samples from SARS-CoV-2 positive patients when Ct value was below 25.

The number of dogs in our training protocol was limited to 6, while we started with 13 at the beginning, as a number of dogs did not pass the initial selection tests. This corresponds to the range typically used in other studies, where the use of 6–12 dogs is common (13, 14, 24–26). The performances of the 6 Belgian dogs exhibited similarity to that observed many other studies using armpit sweat samples.

During the training phase, the dogs performed 6 to 10 runs per day, 3 to 4 days per week, representing an intensive regimen designed to enhance their learning capacity. Careful consideration was given to prevent olfactory fatigue and performance decline by avoiding excessive runs that may induce exhaustion. In the validation phase, the dogs received training to 5 days per week, while after validation, the training frequency reduced to 1 day every 2 weeks. Consequently, the dogs were more relaxed, although the overall performances after validation were not significantly different compared to the validation phase.

Proper sampling methods are crucial for training effective detection dogs. It is essential to use samples that have been confirmed positive or negative through qPCR testing. Sampling time with the patients was a determining factor in successful identification by the detection dog. In our trial, samples from each patient/participant were transferred into plastic bags, frozen to −20°C, transported, stored for some time, thawed (30 min) (−20°C to 16°C) before being presented to the detection dog. However, this process can result in a certain loss of volatile molecules. To mitigate this loss, we aimed to ensure a minimum contact time between sample and armpit of 15 min, with the majority of samples having a contact time of 30 min. In contrast, other studies have generally employed shorter contact times (12–14, 20–22). Based on our experience, a longer initial sampling time, allowing for greater retention of odorous molecules in the cotton pads, is preferred to account for the losses during freezing, thawing, transportation and storage (27). In our study design, a sampling time of 30 min is preferred over 15 min. However, some studies have demonstrated good results with a contact time as short as 5 min (20), where samples were provided to the dogs fresh or stored at +4°C (21). In our study, all samples were stored at −20°C, as working with fresh samples risked evaporation of volatiles between sampling and training. Increasing the contact-time between cotton balls and armpit may improve the quality of the sample when freezing is involved, but it may not be practical in field conditions. The possible solution is to use fresh samples for testing, which would reduce the contact time between cotton balls and patients while maintaining good performances of the test.

During the validation phase, a rigorous approach was employed to ensure the reliability of the results. Unlike other studies, no empty cones devoid of human scent (blank) were used, but sweat samples worn by confirmed SARS-CoV-2 positive or negative cases were exclusively employed. Additionally, the dogs were exposed to completely negative series, consisting of samples coming from different volunteers. This rigorous method was not found in previous studies and was superior to other studies conducted on sweat samples. The validation process was undertaken in double-blinded conditions. To prevent any sampling center-related bias, sweat samples were obtained from different locations, from patients with different clinical signs, and at different moments of the day, irrespective of the Ct-value. This comprehensive approach aimed to ensure the robustness of the results by avoiding any potential biases associated with the sampling center.

At the beginning of the training, cotton balls and gauze were used as scent carriers. In order to enable the dogs to discriminate the specific SARS-CoV-2 odor, it was essential to consistently present the same scent carriers for positive or negative samples. Almost all negative samples were collected on cotton balls so it was necessary to collect the positive samples on cotton balls as well. Otherwise, the dogs could learn to distinguish cotton from gauze, which is not wanted.

Variations in the performance of the dogs may come from a range of different factors, including their individual skills, specific function (explosive detection versus search and rescue), conditioning, training, age, diet and environment (28). In our study, we observed a potential lower performance when the outside temperature was higher than 25°C, despite conducting training sessions in a controlled environment with regulated temperature and humidity. The elevated temperatures could exhaust the dogs in between the runs. It is known that dehydration of the mucosal layer in the canine nasal cavity can significantly decrease odor detection capabilities (29, 30).

During the training phase, the dogs had the tendency to show hesitation at both the first and last cones. In future training setups, it would be preferable to arrange the cones in a circular way to address this issue. In order to improve the sensitivity of the dogs, double validation (2 dogs testing the same sweat samples) could be performed on real samples. Previous studies have reported a sensitivity of up to 100% using this double validation (22). The specificity of the 6 trained dogs was overall very high. This is an interesting characteristic because it allows SARS-CoV-2 negative people to not be stuck in an airport or other place/event in case of a false positive result. Despite a high specificity, confirmed negative patients did not have another qPCR test several days later to confirm the result.

There was no significant correlation found between the age, biological gender, body mass index, or deodorant use of the participant that provided the sample and the detection (or hesitation) by the trained dogs. This is good news, as this means that deodorants cannot cover up an underlying SARS-CoV-2 infection of a patient. Similarly, individual characteristics such as biological gender, age and body mass index do not influence the efficacy for trained dogs to detect an underlying infection. This makes the detection dog method a robust and uniform detection method for SARS-CoV-2.

The trained SARS-CoV-2 dogs correctly identified 100% of vaccinated people as negative, and thus healthy. The individuals received the Comirnaty^®^ vaccine in two doses and were sampled 3 weeks after the second dose. At least after 3 weeks, the vaccination process did not interfere with the dogs’ ability to distinguish positive and negative samples, thus avoiding false positive detection in healthy vaccinated people.

The trained detection dogs were able to detect a mixture of different volatiles (Figure 3; Table 3; Supplementary Figure S4). Particular volatiles were repeatedly retrieved and significantly associated with SARS-CoV-2 positive sweat samples, and not associated with SARS-CoV-2 negative sweat samples. Some of the volatiles were breakdown compounds and could be traced back. 1-octen-3-ol, detected in higher abundances in SARS-CoV-2-positive sweat samples, is known to be secreted by human skin and is an important attractant for mosquitoes (31). It is a breakdown product of linoleic acid, which was identified as an important antiviral fatty acid. A study showed that linoleic acid was the most antiviral against the SARS-CoV-2 virus, with a direct binding to the cavity formed by the RNA double helix and protein (32). As such, it is hypothesized that 1-octen-3-ol is present in sweat in higher amounts because of the activity of linoleic acid. These alcohols have been found in the breath of (critically ill) SARS-CoV-2 patients (19, 33–35) which confirms their elevation with SARS-CoV-2 infection. DL-3,4-dihydroxymandelic acid and its derivatives are metabolites of norepinephrine and have antioxidant properties (36). Norepinephrine is an important hormone and neurotransmitter in the human body which is released in higher levels during situations of stress or danger (37). Norepinephrine is also a known neurotransmitter in the Merkel cells located in the skin (38). Norepinephrine (or a structurally related neurotransmitter) may be implied in the cytokine storms present in SARS-CoV-2 patients (39, 40). In that case, the dogs can detect the neurotransmitter metabolites that are implied in the cytokine storms of SARS-CoV-2 patients. Several volatiles related to urocanic acid were detected in higher abundances in SARS-CoV-2-positive samples. Urocanic acid is naturally present in human sweat and in the stratum corneum and is a breakdown product of filaggrin (41). It is known to act as a photo protectant and absorbs UVB light (41). It could be upregulated in SARS-CoV-2-positive sweat samples as part of the natural immune reaction of the human body to the Sars-CoV-2 virus, although the real reason remains to be elucidated. Octadecyl acetate was similarly repeatedly found and associated with SARS-CoV-2 positive sweat samples. It remains unclear why these volatiles are upregulated. A variety of other volatiles have been identified (Table 3) and all add up to the unique scent associated with infection that the detection dogs were able to pick up.

Detection dogs are widely used to detect narcotics and explosives (42), however, to the public, it is relatively unknown that detection dogs can be used to detect SARS-CoV-2 infection in humans. The Belgian population largely supports the SARS-CoV-2 detection dogs as a valid detection technique however; a minority of people raised some constraints (Supplementary Figure S5). The use of a quick antigen test seems to be easier to handle compared to detection dogs (43). Some respondents were afraid of dogs but this can be solved by avoiding direct contact between a screened person and a detection dog. Communication on the possibilities of SARS-CoV-2 detection dogs was very important in order to increase overall acceptance as a valid SARS-CoV-2 detection test. Nonetheless, based on our large Belgian survey, most of the Belgian people were open toward the use of detection dogs to detect SARS-CoV-2.

In general, people were more open toward detection by a dog, rather than taking a nasal sample for qPCR test. The latter is more invasive as compared to taking a sweat sample (44). Respondents also preferred to provide an armpit sweat sample, rather than socks, urine, shirt, or neck sweat sample. Providing an armpit sweat sample is one of the least invasive methods to detect on SARS-CoV-2, together with providing a disposable mouth mask and saliva. The Belgian respondents saw great value in using the detection dogs at the airport. Other preferred locations were cultural and sports events. Detection dogs would indeed be very useful in these locations, where large numbers of people can be screened in a small amount of time and with minimal efforts (45). The main reservations that people had toward such detection dogs were related to ethical considerations, safety and organizational considerations. Similar ethical considerations have been noted before (19).

The current study has limitations. Ideally, the dogs can also detect asymptomatic people, but we had only few of these samples during training. The main objective was to train detection dogs on the ability to distinguish between SARS-CoV-2 infected and healthy people, and we therefore primarily trained with positive samples coming from patients with clear symptoms and qPCR results of <25 cycles.

We did not confirm the absence of SARS-CoV-2 virus in the dogs’ nose after training as the probability was very low. There was no direct skin contact between the sample and the dog’s nose. It is also well known that sweat is not a classical way of virus excretion. Finally, the virus replicates very rarely in dogs (46), and only after prolonged and direct contact with a highly contagious patient. Therefore, the chance of viral transmission is minimal. Dog trainers were tested regularly and were not tested as positive. In addition, no dog or trainer got ill during the training, validation or post-validation.

The Belgian government supported the training of our SARS-CoV-2 detection dogs and acknowledged the great results obtained. However, the government wished to deploy the dogs directly among a crowd of people, which required a more specific training. Since the vaccines and the quick antigen tests were widely available, the Belgian government did not further support the detection dogs’ program.

## Conclusion

Detection dogs can be efficiently trained to detect SARS-CoV-2 based on sweat samples obtained from the armpit. The trained dogs exhibit a high specificity, rarely indicating a negative sample as positive. Ensuring the collection of high-quality samples is crucial, involving a consistent sampling protocol, with the use of the same carrier and sufficient odor captured. An appropriate storage at cold temperature (4°C or − 18°C or − 20°C) and 30 min sampling time is preferred, accompanied by confirmation of the sample being positive or negative through qPCR testing. Positive samples from symptomatic patients and samples sourced from different hospitals are recommended to avoid center bias.

A training protocol was constructed whereby the goal was to have 6 to 10 runs per day per dog. This range was determined to be sufficient for effective training, as fewer runs were insufficient to train the dogs, while more runs resulted in dog exhaustion. Additionally, detection dogs which were pre-trained to detect ‘in line’ were found to be easier for the training on SARS-CoV-2 samples. The dogs detected a wide variety of volatiles, among which a series of breakdown compounds of antiviral fatty acids and neurotransmitters/hormones.

The general public demonstrated a high acceptability toward the utilization of canines as SARS-CoV-2 detection tools. However, it is crucial to establish a proper practical setup. Direct contact between screened person and detection dog should be avoided, to deal with the fear that some people have. Enhancing overall acceptability requires effective communication regarding the possibilities and efficacy of the SARS-CoV-2 detection dogs. Sampling of the armpit for this purpose is preferred over a nasal swab for qPCR test, as it offers a less invasive approach.

## Data availability statement

The raw data supporting the conclusions of this article will be made available by the authors, without undue reservation.

## Ethics statement

The studies involving animals were reviewed and approved by the University of Liege (approval number N°20-2246). The studies involving human participants were reviewed and approved by Ethical Committee UZ Gent, approval number multicentric study BC-08571, as well as all participating hospitals (listed under Materials & Methods). The patients/participants provided their written informed consent to participate in this study. Written informed consent was obtained from the individual(s) for the publication of any identifiable images or data included in this article.

## Author contributions

CC, MS, FG, and HG designed the experiments. MP, RV, BaM, EV, PV, CC, and HG did the metadata collection. MP, RV, BaM, EV, and PVG trained the detection dogs and did the experimental organization and setup. AP, BeM, GD, SP, LDV, FS, KVV, SoT, AO, IM, PV, SJ, LV, ET, GW, J-CM, KA, LD’H, SeT, BDT, and JC collected samples from patients. AL, AM, and AA did the GC/MS analysis and putative identification of volatiles. DG provided the initial dog training and supplied feedback. CC and HG wrote the manuscript. CC made the figures and did the statistical analysis. All authors critically revised the manuscript for important intellectual content.
