# Supplementary material for: Sniffing out safety: canine detection and identification of SARS-CoV-2 infection from armpit sweat
Source: Front Med (Lausanne). 2023 Sep 19;10:1185779. doi: 10.3389/fmed.2023.1185779 (PMC10563588; doi:10.3389/fmed.2023.1185779)
Supplement: Supplementary file 1 [file Data_Sheet_1.pdf]

Sniffing Out Safety: Canine Detection and Identification of SARS-CoV-2 Infection from Armpit Sweat Supplementary information

Doi: 10.3389/fmed.2023.1185779

Supplementary text

Acceptability results - supplementary information

Would you trust the corona dogs test result?

Only 4.6% of the responders would (maybe) not trust the outcome of the SARS-CoV-2 detection dogs (**Figure 1B**). This is an important confounding factor in the general acceptance of the general public towards SARS-CoV-2 detection dogs (Spearman correlation analysis rho=0.4214385; Z = 25.174, $p < 0.001$).

The main confounding factor was that the responders had more trust in the qPCR test, as compared to the SARS-CoV-2 detection dog outcome (Spearman correlation analysis rho=0.5131067; Z = 30.602, $p < 0.001$).

About 71% of the responders that have no trust in the detection dog outcome do not accept the use of SARS-CoV-2 detection dogs, while 91% of the responders that have absolute trust in the dogs, also fully accept this method.

Ethical constraints.

3.2% of the responders found it absolutely unethical to use dogs for this purpose. The ethical concerns were the major confounding factor in the general acceptance of the corona dogs in practice (Spearman correlation analysis rho=0.6728146; Z = 23.702, $p < 0.001$). About 71% of the responders that said it is very unethical to use dogs for the detection of SARS-CoV-2, also do not accept this method. While 95% of the responders that have no ethical problems with the use of detection dogs completely accept the use of detection dogs to trace SARS-CoV-2. It was also a main confounding factor in the general trust in the outcome of the SARS-CoV-2 detection dogs (Spearman correlation analysis rho=0.3981205; Z = 14.025, $p < 0.001$).

Practical organization.

These practical doubts were also a main confounding factor in the general acceptance of the corona dogs in practice (Spearman correlation analysis rho=0.4990985; Z = 17.547, $p < 0.001$). About 57% of the responders that said it is very difficult or impossible to organize the detection of SARS-CoV-2 with dogs, also don't accept the use of dogs for the purpose of detecting SARS-CoV-2 infection with people. It was similarly a main confounding factor in the general trust in the outcome of the SARS-CoV-2 detection dogs (Spearman correlation analysis rho=0.4108794; Z = 14.451, $p < 0.001$).

Safety to use dogs. Fear of dogs.

The large majority (74.8%) of the responders did not find it dangerous at all to use dogs for the purpose of detecting SARS-CoV-2 infection with people. There were however still some doubts on the safety of using sniffer dogs and it was the second biggest confounding factor in the general acceptance of SARS-CoV-2 detection dogs in practice (Spearman correlation analysis rho=0.6488901; Z = 22.877, p < 0.001). About 64% of the responders that said it is very dangerous to use dogs for the detection of SARS-CoV-2, also do not accept the use of sniffer dogs in practice. It was similarly the second biggest confounding factor in the general trust in the outcome of the SARS-CoV-2 detection dogs (Spearman correlation analysis rho=0.442513; Z = 15.595, p < 0.001).

We asked whether the people were afraid of dogs: about 73.7% indicated that they were not afraid of dogs at all. 9% of the people indicated to be afraid of dogs (of which 2% were very afraid of dogs) and another 17.3% might be a little afraid of dogs. Fear of dogs was a confounding factor in the general acceptability (Spearman correlation analysis rho=-0.101809; Z = -6.0839, p < 0.001). 79% of the responders that do not fear dogs at all fully accept the use of SARS-CoV-2 detection dogs, while only 61% of the responders that are very afraid of dogs fully accept this. It was similarly a confounding factor in the general trust of the outcome of the detection dog (Spearman correlation analysis rho=-0.1851397; Z = -11.06, p < 0.001).

We also asked whether the people were allergic to dogs: the large majority (91%) was not allergic to dogs at all, while 9% of the responders were a little or a lot allergic to dogs. We asked whether the people had religious problems with dogs: only 1.1% of the responders had some sort of religious problem with dogs in Belgium. These latter two factors did not have any significant relationship with the general acceptability in the SARS-CoV-2 detection dogs (Spearman correlation analysis respectively rho=-0.005947897; Z = -0.21071, p = 0.8385 and rho=0.01111675; Z = 0.39335, p = 0.6636).

Willingness to give their armpit sweat to train SARS-CoV-2 detection dogs.

The willingness to share their armpit sweat to train detection dogs was also a confounding factor in the acceptance of the responders (Spearman correlation analysis rho=0.3802234; Z = 22.693, p < 0.001). About 53% of the people that didn't want to share their sweat to train detection dogs, also disapproved of the use of detection dogs to detect SARS-CoV-2 infection. This was also a confounding factor in the general trust in the outcome of the detection dogs (Spearman correlation analysis rho=0.3807756; Z = 22.719, p < 0.001).

However, when asking which sample the responders would be most likely to give for the SARS-CoV-2 detection dog to evaluate, the majority indicated they would rather give a sample of their armpit sweat (64.2% of the responders) than a nasal sample (24.3% of the responders). Other samples that the responders are willing to provide are their mouth mask (70.0% of the responders) and a sample of their saliva (66.9% of the responders). For the purpose of the dog test, responders are less willing to provide their socks (45.3% of the responders), urine (41.5% of the responders), their shirt (40.8% of the responders) or sweat from their neck (40.7% of the responders).

Communication on the SARS-CoV-2 detection dogs was of influence in the general acceptability and trust in the detection dogs. 82.5% of the responders had heard or read about the SARS-CoV-

2 detection dogs, while 17.5% had not heard about these detection dogs before filling in the survey. The overall acceptability toward the use of SARS-CoV-2 detection dogs increased if the responders had already heard or read about the possibilities of the SARS-CoV-2 detection dogs (Spearman correlation analysis rho=-0.2670125; Z = -15.961, p < 0.001). Communication in the press and social media resulted in a 30% increase toward acceptability in the use of detection dogs to detect SARS-CoV-2 in practice. Communication also had a significant impact on the general trustworthiness of the SARS-CoV-2 detection dogs (Spearman correlation analysis rho=-0.2906706; Z = -17.37, p < 0.001). Only 20.7% of the responders that never heard or read about them had absolute trust in the SARS-CoV-2 detection dog outcome, while this more than doubled to 50.2% for the responders that did read or hear about it in the news or social media.

The ***age*** of the responders also had a significant impact on the overall acceptability and trust in the SARS-CoV-2 detection dogs. The younger the age group, the less acceptive the responders were regarding the use of SARS-CoV-2 detection dogs (Spearman correlation analysis rho=-0.09428571; Z = -5.6367, p < 0.001). Only 58% of the responders younger than 20y fully support the use of SARS-CoV-2 detection dogs. This increases to 74% of the responders aged 21-40y old, and again increases to 79% for the responders aged 41-65y. The most acceptability (85%) was seen for the responders aged older than 65y. The age of the responders also had a significant impact on the general trustworthiness of the outcome of the detection dogs (Spearman correlation analysis rho=-0.13904; Z = -8.3099, p < 0.001). Younger people were less likely to trust the dog's outcome (58% for people <20y), as compared to older people (85% of the people >65y). The age similarly had an impact on the question whether the responders would accept being refused at the border or entrance of an event by the result of SARS-CoV-2 detection dogs (Spearman correlation analysis rho= -0.1221055; Z = -7.2978, p < 0.001). Only 32% of the people younger than 20y fully supported being refused at the border or entrance based on the outcome of the coronadog. The support base gradually increased with age, to 63% of the older generation (>65y) fully supported being refused at the border based on the dog's outcome.

About 84.3% of the people had a ***pet at home***, while 15.4% of the people did not have any pets at home. Having pets at home did not have a significant correlation with the general acceptability toward the SARS-CoV-2 detection dogs (Spearman correlation analysis rho=-0.02507726; Z = -1.4973, p = 0.1268). It did however have a significant impact in the general trust in the outcome of the detection dogs (Spearman correlation analysis rho=-0.08460813; Z = -5.0503, p < 0.001). It similarly had an impact on the general acceptance of being refused at the border or entrance of an event, by the outcome of SARS-CoV-2 detection dogs (Spearman correlation analysis rho= -0.07388211; Z = -4.4101, p < 0.001). Those that had pets at home were 10% more likely to fully support the idea of being refused at the border based on the dog's outcome.

We asked in the questionnaire whether they would accept ***being refused at the border*** or entrance of an event by the result of SARS-CoV-2 detection dogs. About 45.1% of the responders indicated that they would have no problems being refused at the border or entrance on the basis of the SARS-CoV-2 detection dog outcome. There is still support for this, but the support is considerably smaller as compared to the general acceptability of SARS-CoV-2 detection dogs. Major reasons (and significant correlations) are largely attributed to the distrust in the corona dog

outcome (Spearman correlation analysis rho=0.5793072; Z = 34.594, p < 0.001); the disagreement to use dogs for the purpose of detecting SARS-CoV-2 with people (Spearman correlation analysis rho=0.3679556; Z = 21.979, p < 0.001); the difficult organization to use detection dogs in practice (Spearman correlation analysis rho= 0.3314874 ; Z = 11.663, p < 0.001); the fact that would is dangerous to use dogs for this purpose (Spearman correlation analysis rho= 0.3303303 ; Z = 11.642, p < 0.001); the fact that responders had more trust in the qPCR test outcome as compared the corona dog outcome (Spearman correlation analysis rho= 0.3258414; Z = 19.433, p < 0.001); the fact that it is unethical to use dogs for this purpose (Spearman correlation analysis rho= 0.3217696; Z = 11.335, p < 0.001); the fact that responders did not want to give their sweat (Spearman correlation analysis rho= 0.3170247 ; Z = 18.921, p < 0.001); the fact that people had not yet heard or read about the possibilities of the corona dogs (Spearman correlation analysis rho= -0.1968382; Z = -11.763, p < 0.001) and the fact that responders were afraid of dogs (Spearman correlation analysis rho= -0.1322548 ; Z = -7.901, p < 0.001).

When we asked about the location where to deploy the corona dogs, the responders preferred to use them in the airport (88.4% of the responders). Other preferred locations were cultural events such as concerts and music gatherings (78.0% of the responders), and sports events (70.9% of the responders). Other less preferred locations were schools and universities (62.7%), by medical and civil protection personnel (57.0%), elderly homes (55.2%) and conferences (50.7%). Least preferred locations, as indicated by the responders, were their workplace (33.6%), their hotel (30.6%), or their homes (14.3%).

About 63% of the Belgian population spoke Dutch (2261 of the 3391) and 37% spoke French (1330 of the 3391), representing the different language groups in Belgium (Dutch in Flanders, French in Wallonia and both in Brussels). The language group did not have any influence in the general acceptability or trust in the SARS-CoV-2 detection dogs. Three quarters of the people that filled in the survey were female (75.3% - or 2704 of the 3591). The biological gender, however, did not have a significant correlation with the general acceptance on the use of SARS-CoV-2 detection dogs (Spearman correlation analysis rho=0.02664743; Z = 1.5859, p = 0.11), nor in the general trust in the outcome of the detection dogs. About 62.2% of the responders claimed to not have contracted SARS-CoV-2 before, 18.3% of the responders didn't know where they have contracted it before, 7.7% of the responders probably had SARS-CoV-2 but it wasn't tested for, and the remaining 11.8% indicated that they had contracted SARS-CoV-2 prior to filling in the survey. This did not have a major impact on the acceptability of the SARS-CoV-2 detection dogs (Spearman correlation analysis rho=-1.752227e-05; Z = -0.0010475, p = 0.9987). The majority of the responders (62.2%) did not work in a medical environment (veterinary doctor, medical doctor, pharmacists, nurses, etc). These people were more likely to have more trust in the qPCR outcome as compared to the SARS-CoV-2 detection dog outcome. About 9% of the responders (332 people) indicate that they work with detection dogs; and these people had considerably more trust in the SARS-CoV-2 detection dog outcome, as compared to the qPCR result. These professions further had no major impact in the general acceptance on the use of SARS-CoV-2 detection dogs.

Survey questionnaire.
Which language do you speak? French or Dutch.
What is your biological gender? Female, male or other.
What is your age? 0-20y; 21-40y; 41-65y; >65y.
Do you have animals at home? Yes; No.
Do you work in a medical environment (veterinary, doctor, pharmacist, nurse, ...)? Yes; No.
Do you work with detection dogs (police, army, fire squad, civil protection, at home, training dogs, …)? Yes; No.
Are you afraid of dogs? Scale 1->5
Are you allergic to dogs? Scale 1->5
Do you have religious problems with dogs? Scale 1->5
Did you know that dogs can detect COVID19 infection with humans based on sweat odor? Yes; No.
Did you ever have COVID19 infection? Yes; No; Don't know; Probably, but without test.
Do you agree that dogs can be used to diagnose COVID19 infection? Scale 1->5
Do you think that it is safe to use dogs to diagnose COVID19 infection from armpit sweat? Scale 1->5
Do you think it is ethical to use dogs to diagnose COVID19 infection? Scale 1->5
Do you think it is organizationally possible to use dogs to diagnose COVID19 infection in practice? Scale 1->5
Where in daily life could the COVID19 detection dogs be used? Airport/station/port; Schools & universities; Medical personnel/civil security/police/army; Nursery homes; Sports events; Cultural events/festivals; Conferences; Workplace; Hotels; At your home.
Do you agree to test on COVID using a dog, based on your sweat sample? Scale 1->5
Do you agree that, based on a COVID test done by a dog, it is prohibited to enter a country, event, sports, other? Scale 1->5
Would you allow to give your sweat to train corona dogs? Scale 1->5
Which sample do you prefer to give to verify presence at Covid19? Armpit sweat; urine; mask; T-shirt; saliva; nasal swab; socks.
Would you trust the result of the corona dog? Scale 1->5
Would you trust the corona dog test more than the PCR test? Scale 1->5
Upon arrival in the airport, which test would you prefer as corona test? Test armpit sweat by dog; Test nasal sample with qPCR; both of the above; another fast test.
Other remarks on the use of COVID19 detection dogs?

Supplementary tables

Table S1. Raw data presenting the individual dog's test responses to human sweat samples presentation. The column "+" indicates a positive SARS-CoV-2 sample while the column "-" indicates a negative one. The line "yes"/column"+" indicates the number of samples that dogs consider as positive (e.g. immobilization of the dog, showing to the owner that his dog has found a SARS-CoV-2 infected human) and that are really infected (true positive); the line "no"/column"+" indicates the number of samples that dogs consider as negative while they are infected (false negative). The combination "yes"/"-" and "no"/"-" represent the false positive and true negative, respectively.

	Dog's test response	COVID SAMPLES INFECTION STATUS		Total
		+	-	
Lily	Yes	60	0	60
	No	19	292	311
	Total	79	292	371
Xhena	Yes	49	10	59
	No	3	230	233
	Total	52	240	292
Tina	Yes	45	13	58
	No	17	248	265
	Total	62	261	323
Paxy	Yes	50	0	50
	No	7	243	250
	Total	57	243	300
Bailey	Yes	55	1	56
	No	17	306	323
	Total	72	307	379
Chaeos	Yes	61	3	64
	No	14	283	297
	Total	75	286	361
**TOTAL**	Yes	320	27	347
	No	77	1602	1679
	Total	397	1629	**2026**

Table S2. Contingency table and formulas used to calculate the performances of the detection dogs in diagnosing SARS-CoV-2 from human sweat samples in the validation and post-validation phase (according to (46)**).**

		INFECTION STATUS		
		**Positive**	**Negative**	
**TEST RESPONSE**	**YES**	**TP**	**FP**	**TP + FP**
	**NO**	**FN**	**TN**	**FN + TN**
		**TP + FN**	**FP + TN**	**Total**

**Se :**	**TP/(TP+FN)**	**Sp :**	**TN/(TN+FP)**
**PPV :**	**TP/(TP+FP)**	**NPV :**	**TN/(TN+FN)**
**Accuracy :**	**(TP+TN)/(TP+TN+FP+FN)**	**Youden : index**	**Se + Sp - 1**

Caption: Se: Sensitivity; Sp: Specificity; TP: true positive; FP: false positive; FN: false negative; T: true negative; PPV: positive predictive value; NPV: negative predictive value.

Table S3. Comparative performances of the dogs with samples of vaccinated people (n=28, Comirnaty, BioNTech-Pfizer, 2 doses, samples 3 weeks after 2nd dose) mixed with negative and positive SARS-CoV-2 samples. If only Negative (vaccinated) Samples are considered, then results are **100%** successful for the 5 dogs.

DOG	Se*	Sp*	PPV*	NPV*	Youden	n* Run
Paxi	75	100	100	89	75	2
Cheos	100	100	100	100	1	1
Xhena	83	100	100	92	83	3
Lilly	63	100	100	84	63	4
Bailey	100	100	100	100	1	1
**TOTAL**	**77**	**100**	**100**	**90**	**77**	**10 ± 2**

* Se: Sensitivity; Sp: Specificity; PPV: positive predictive value; NPV: negative predictive value; n: number.

Supplementary Figures

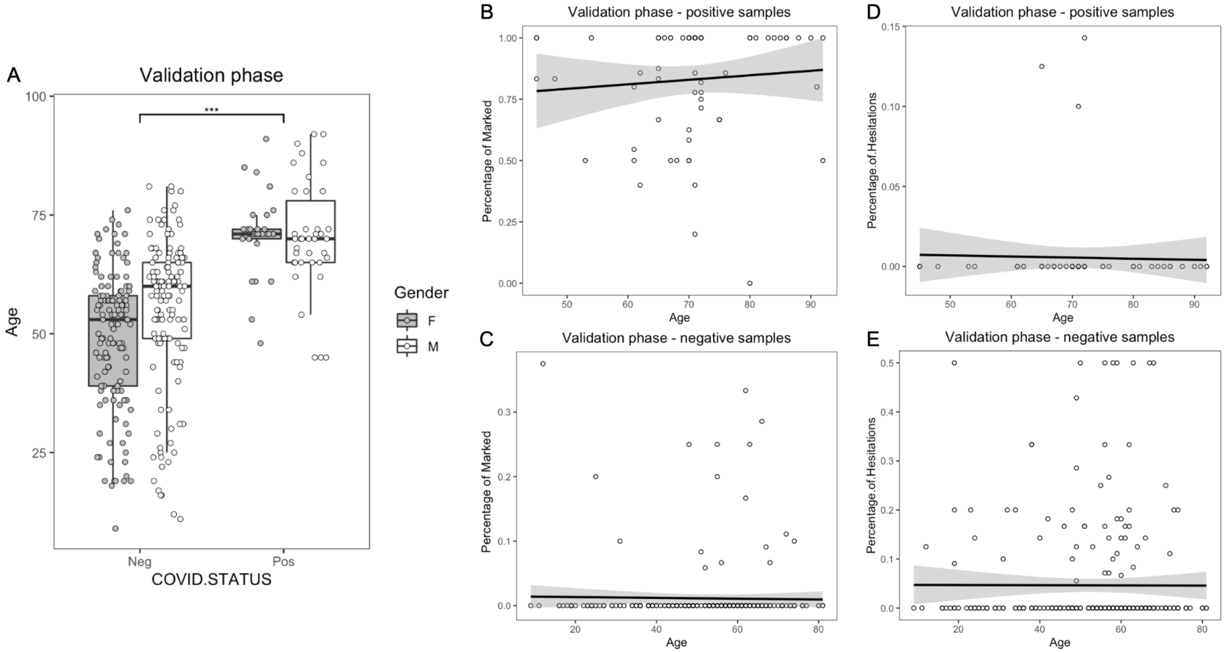

Figure S1: Impact of age of the patient or volunteer on marking by the six trained SARS-CoV-2 detection dogs during validation phase. A/ Distribution of age of participant per biological gender group in the SARS-CoV-2-positive and -negative group. B/ Impact of age on percentage of marking by the detection dogs in the SARS-CoV-2 positive samples (from hospitals): no significant effect (p>0.05). C/ Impact of age on percentage of marking by the detection dogs in the SARS-CoV-2 negative samples (from volunteers): no significant effect (p>0.05). D/ Impact of age on percentage of hesitations by the detection dogs in the SARS-CoV-2 positive samples (from hospitals): no significant effect (p>0.05). E/ Impact of age on percentage of marking by the detection dogs in the SARS-CoV-2 negative samples (from volunteers): no significant effect (p>0.05).

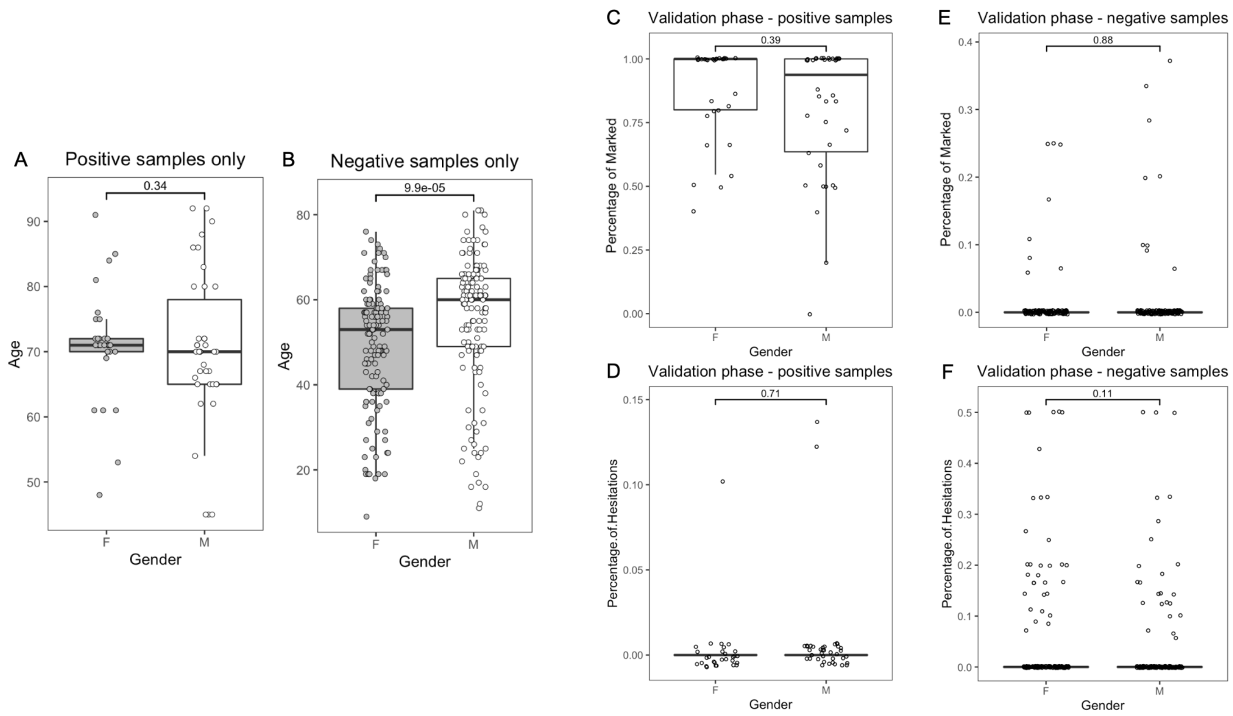

Figure S2: Impact of biological gender of the patient or volunteer on marking by the six trained SARS-CoV-2 detection dogs during validation phase. A/ Distribution of age of participant per gender group in the SARS-CoV-2-positive group: no significant difference. B/ Distribution of age of participant per gender group in the SARS-CoV-2-negative group: significant difference. C/ Impact of gender on percentage of marking by the detection dogs in the SARS-CoV-2 positive samples (from hospitals): no significant effect. D/ Impact of gender on percentage of hesitations by the detection dogs in the SARS-CoV-2 positive samples (from hospitals): no significant effect. E/ Impact of gender on percentage of marking by the detection dogs in the SARS-CoV-2 negative samples (from volunteers): no significant effect. D/ Impact of gender on percentage of hesitations by the detection dogs in the SARS-CoV-2 negative samples (from volunteers): no significant effect.

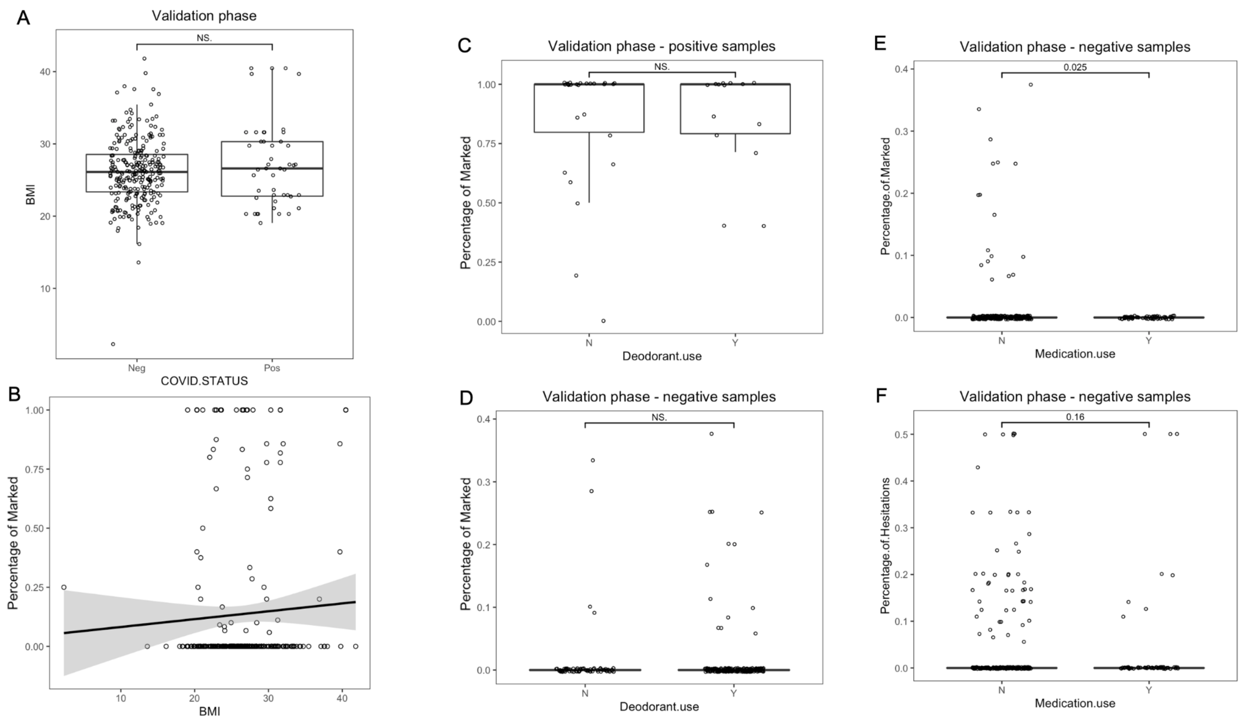

Figure S3: Impact of body mass index (BMI), deodorant use and medication use of the patient or volunteer on marking by the six trained SARS-CoV-2 detection dogs during validation phase. A/ Distribution of BMI of participant per gender group in the SARS-CoV-2-positive and -negative group: no significant difference is noted. B/ Impact of BMI on percentage of marking by the detection dogs in the validation phase: no significant correlation (p=0.6156). C/ Impact of deodorant use on percentage of marking by the detection dogs in the SARS-CoV-2 positive samples (from hospitals): no significant effect. D/ Impact of deodorant use on percentage of marking by the detection dogs in the SARS-CoV-2 negative samples (from volunteers): no significant effect. E/ Impact of medication use on percentage of marking by the detection dogs in the SARS-CoV-2 positive samples (from hospitals): significant effect (p=0.025). F/ Impact of medication use on percentage of marking by the detection dogs in the SARS-CoV-2 negative samples (from volunteers): no significant effect (p=0.16).

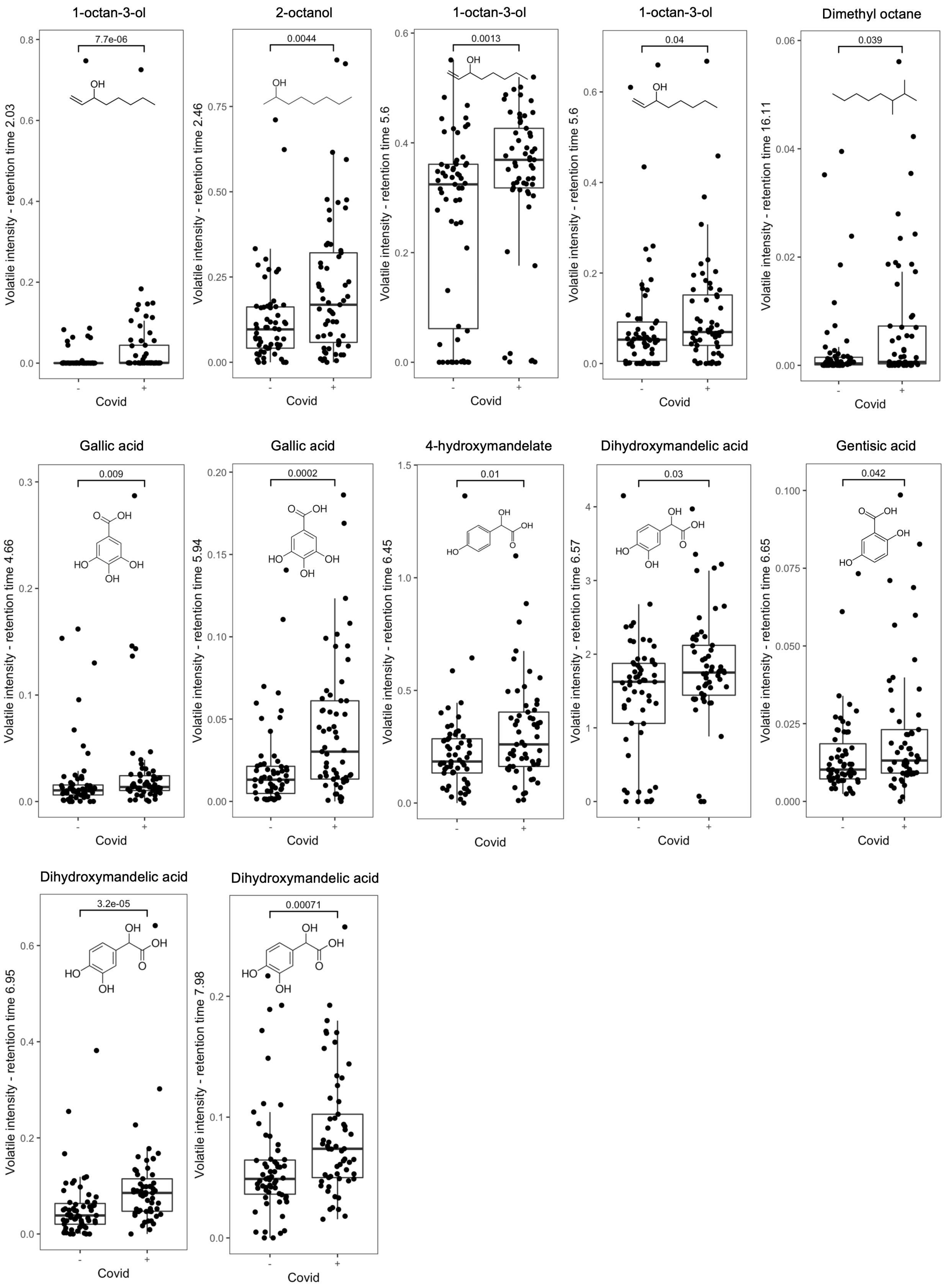
1-octan-3-ol
7.7e-06
Volatile intensity - retention time 2.03
2-octanol
0.0044
Volatile intensity - retention time 2.46
1-octan-3-ol
0.0013
Volatile intensity - retention time 5.6
1-octan-3-ol
0.04
Volatile intensity - retention time 5.6
Dimethyl octane
0.039
Volatile intensity - retention time 16.11
Gallic acid
0.009
Volatile intensity - retention time 4.66
Gallic acid
0.0002
Volatile intensity - retention time 5.94
4-hydroxymandelate
0.01
Volatile intensity - retention time 6.45
Dihydroxymandelic acid
0.03
Volatile intensity - retention time 6.57
Gentisic acid
0.042
Volatile intensity - retention time 6.65
Dihydroxymandelic acid
3.2e-05
Volatile intensity - retention time 6.95
Dihydroxymandelic acid
0.00071
Volatile intensity - retention time 7.98
Covid
-
+

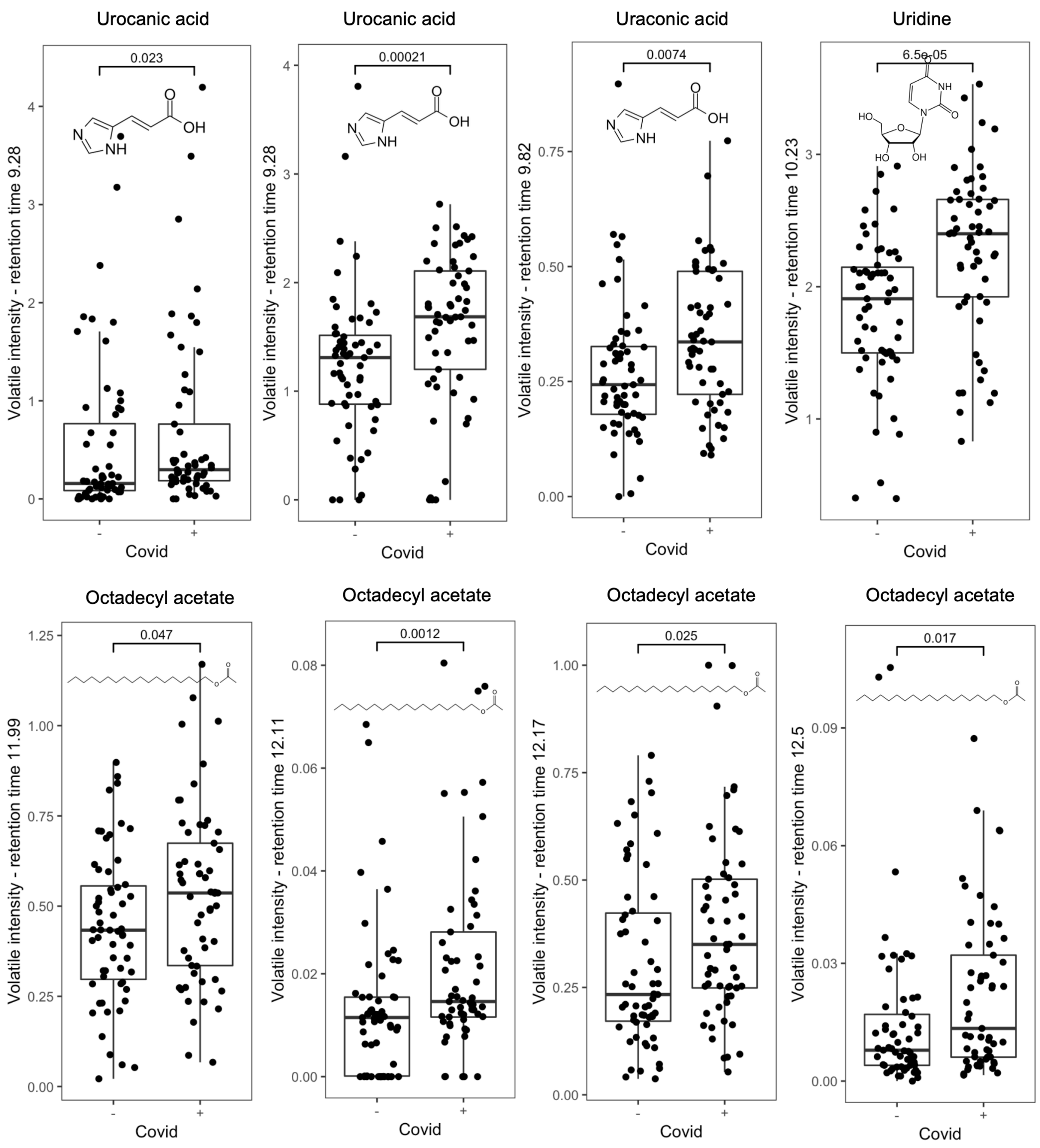

Figure S4. Pairwise boxplot comparison of key volatiles present in confirmed SARS-CoV-2-negative and SARS-CoV-2-positive armpit samples. The y-axis shows the volatile intensity, while the x-axis groups samples per SARS-CoV-2-indication. Significance level is indicated on top. Retention time is shown in the y-axis. Suggested volatile structure is shown in overlay and suggested volatile name is indicated as title. Similar structures are grouped ensemble.

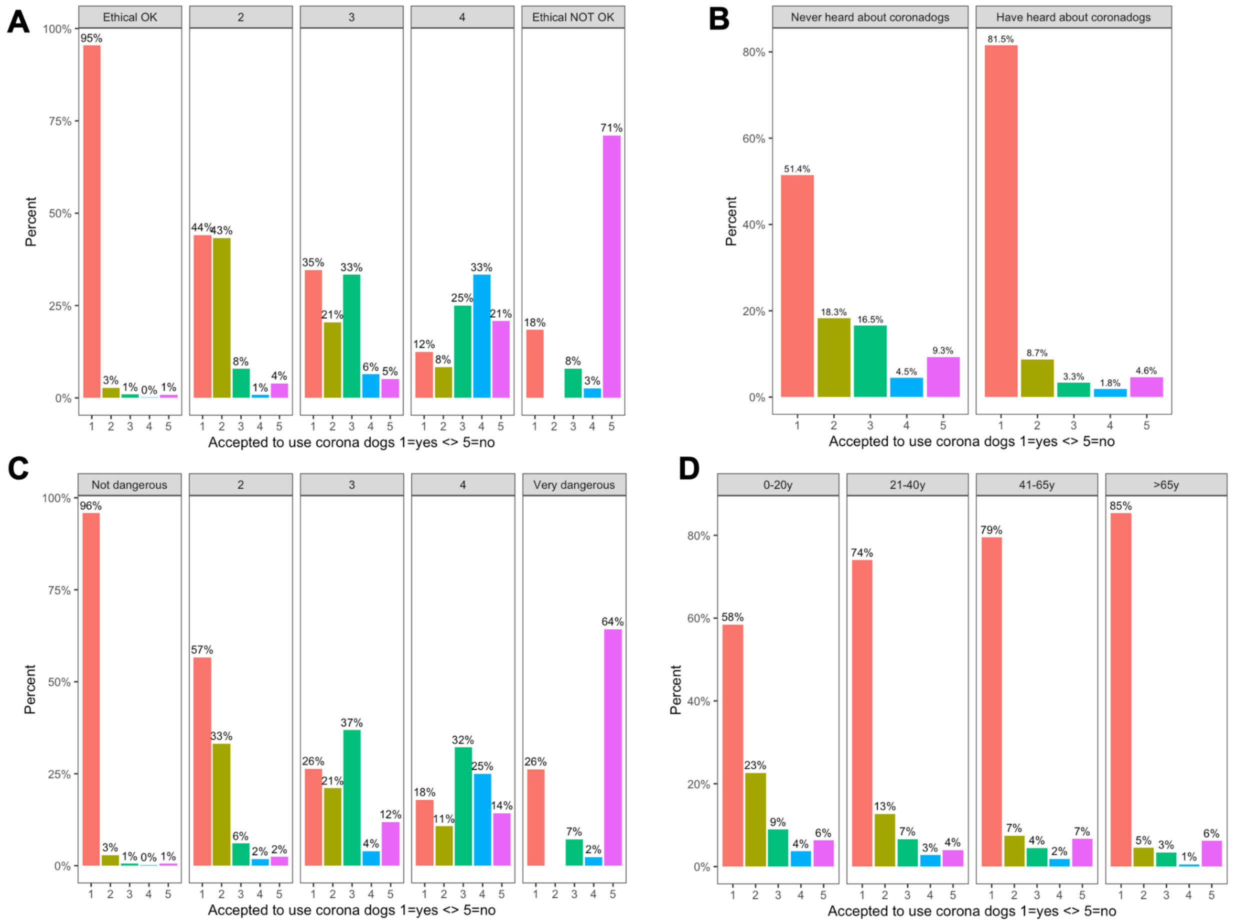

Figure S5. Confounding factors influencing the results on acceptability towards the use of SARS-CoV-2 detection dogs in practice. A/ Ethical constraints on the general acceptability to use detection dogs for the purpose of detecting SARS-CoV-2 infection in people. B/ Impact of communication on the general acceptability. C/ Impact on the fact that people can find it dangerous to use dogs for this purpose. D/ Impact of age on the general acceptability.

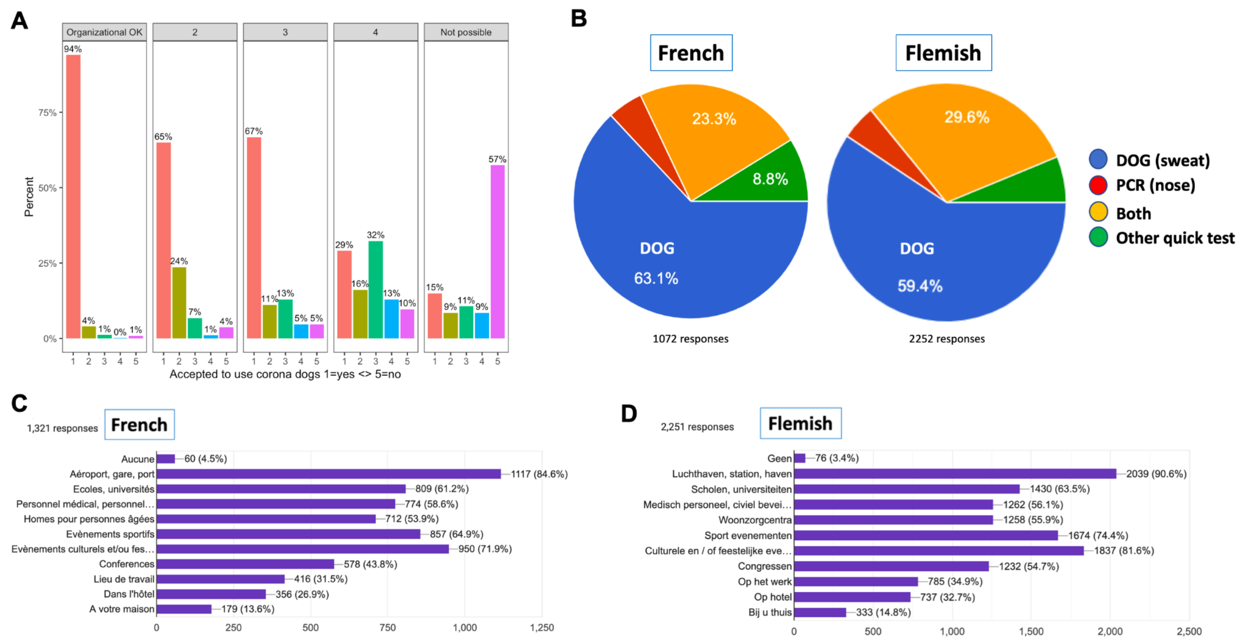

Figure S6. Further results from the national survey on acceptability towards the use of SARS-CoV-2 detection dogs in practice. A/ Organizational constraints on the general acceptability to use detection dogs for the purpose of detecting SARS-CoV-2 infection in people. B/ Which test would you prefer more: the corona dog test, the qPCR nasal test, both tests, or another fast test? C/ Where would you suggest using the detection dogs for this purpose (results from Wallonia)? D/ Where would you suggest to use the detection dogs for this purpose (results from Flanders)?
